# Prenatal tobacco exposure on brain morphometry partially mediated poor cognitive performance in preadolescent children

**DOI:** 10.1515/nipt-2023-0013

**Published:** 2023-07-13

**Authors:** Pedro J. Rodriguez Rivera, Huajun Liang, Amal Isaiah, Christine C. Cloak, Miriam S. Menken, Meghann C. Ryan, Thomas Ernst, Linda Chang

**Affiliations:** Department of Diagnostic Radiology and Nuclear Medicine, University of Maryland School of Medicine, Baltimore, MD, USA; Department of Otorhinolaryngology-Head and Neck Surgery, University of Maryland School of Medicine, Baltimore, MD, USA; Department of Pediatrics, University of Maryland School of Medicine, Baltimore, MD, USA; Department of Neurology, Johns Hopkins University School of Medicine, Baltimore, MD, USA; Department of Neurology, University of Maryland School of Medicine, Baltimore, MD, USA

**Keywords:** cognition, mediation, MRI, preadolescent, prenatal tobacco, sex-difference

## Abstract

**Objectives:**

To evaluate whether prenatal tobacco exposure (PTE) is related to poorer cognitive performance, abnormal brain morphometry, and whether poor cognitive performance is mediated by PTE-related structural brain differences.

**Methods:**

The Adolescent Brain Cognitive Development study dataset was used to compare structural MRI data and neurocognitive (NIH Toolbox^®^) scores in 9-to-10-year-old children with (n=620) and without PTE (n=10,989). We also evaluated whether PTE effects on brain morphometry mediated PTE effects on neurocognitive scores. Group effects were evaluated using Linear Mixed Models, covaried for socio-demographics and prenatal exposures to alcohol and/or marijuana, and corrected for multiple comparisons using the false-discovery rate (FDR).

**Results:**

Compared to unexposed children, those with PTE had poorer performance (all p-values <0.05) on executive function, working memory, episodic memory, reading decoding, crystallized intelligence, fluid intelligence and overall cognition. Exposed children also had thinner parahippocampal gyri, smaller surface areas in the posterior-cingulate and pericalcarine cortices; the lingual and inferior parietal gyri, and smaller thalamic volumes (all p-values <0.001). Furthermore, among children with PTE, girls had smaller surface areas in the superior-frontal (interaction-FDR-p=0.01), precuneus (interaction-FDR-p=0.03) and postcentral gyri (interaction-FDR-p=0.02), while boys had smaller putamen volumes (interaction-FDR-p=0.02). Smaller surface areas across regions of the frontal and parietal lobes, and lower thalamic volumes, partially mediated the associations between PTE and poorer neurocognitive scores (p-values <0.001).

**Conclusions:**

Our findings suggest PTE may lead to poorer cognitive performance and abnormal brain morphometry, with sex-specific effects in some brain regions, in pre-adolescent children. The poor cognition in children with PTE may result from the smaller areas and subcortical brain volumes.

## Introduction

Despite decades of warnings, prenatal tobacco exposure (PTE) remains a significant health issue in the United States. In 2020, 1 in 18 women who gave birth reported smoking during pregnancy [1]. Since PTE exposes the developing fetus to more than 7,000 chemicals (including nicotine and other known carcinogens) [2], smoking during pregnancy may adversely affects fetal brain [3] and cognitive development that persists into childhood (e.g., learning and reading comprehension) [4–7] and adolescence (e.g., memory and attention) [8–10]. However, the evidence remains controversial.

Neuroimaging studies provide additional evidence of structural brain abnormalities following PTE. Children with PTE (ages 6–8 years) had smaller total brain and cortical gray matter volumes, as well as thinning of the superior frontal, superior parietal, and precentral cortices compared to unexposed children [11]. These exposed children also had more affective problems, which were associated with their thinner precentral and superior frontal cortices [11]. Other studies found that compared to unexposed adolescents, those with PTE had smaller amygdalae and thalamic gray matter volumes [12, 13], and the smaller thalamic volumes were associated with enhanced impulsivity [13]. Furthermore, adolescents with PTE had thinner orbitofrontal and middle frontal cortices than unexposed controls [14, 15], with stronger effects in girls than boys [15], suggesting PTE might affect brain development in a sex-specific manner. This is consistent with another task-related functional MRI study where children with PTE who were also current smokers, girls had greater activation in cortical regions involving visual and auditory attention than boys [9]. In addition, in an animal study, prenatal nicotine exposure induced greater downregulation of serotonergic receptors and reduced cholinergic neurotransmission in male than in female rats [16].

Prior studies are often limited by small sample sizes [9, 13–15], included children with common comorbidities (i.e., prematurity) and adolescent tobacco smokers. A recent study using the same cohort as this study only examined the general impact of PTE on overall cognition and global brain measures without considering possible sex-differences [17]. To address these gaps in the literature, we examined children ages 9–10 years from the Adolescent Brain Cognitive Development Study (ABCD), a large and diverse study population, with negligible substance use at this age range [18]. Our objectives were to evaluate whether PTE is related to (i) poorer cognitive performance, (ii) abnormal brain morphometry, and (iii) whether poor cognitive performance is mediated by PTE-related structural brain differences. We hypothesized that compared to unexposed children, those with PTE would have poorer cognitive performance which would be mediated by the smaller brain surface areas, cortical thickness or subcortical volumes in brain regions supporting higher cognitive functions. Lastly, based on prior preclinical and clinical studies of PTE [9, 15, 16], we expected to identify sex-specific PTE effects on cognitive performance and brain measures.

## Subjects and methods

### Data source

We used the ABCD Study baseline dataset from the 2.0.1 data release obtained from the NIMH Data Archive ABCD Collection, which included data from 11,875 children (ages 9–10). All youth were fluent in English, and those born <28 weeks of gestation, or who had a history of severe traumatic brain injury or neurological, developmental, or current alcohol/substance use disorders were excluded [19]. Participants were recruited through a school-based strategy and selected based on sex, race and ethnicity, and socioeconomic status [20], that represented a diverse, national, longitudinal cohort from 21 recruitment sites. The ABCD study recruitment approach and protocol have been described in detail previously [20]. Participants were also primarily drug-naive with minimal substance use, apart from caffeine and less than 1 % had a full drink of alcohol or tried any other substances [18]. Written informed consent from all parents and assent from all children and the research study was approved by a centralized Institutional Review Board at the University of California, San Diego, and the University of Maryland, Baltimore. Participants with non-missing PTE were included in our analyses.

### Prenatal tobacco exposure (PTE)

PTE status was determined from the caregiver-reported developmental history surveys, which asked whether the biological mother used any tobacco products after knowing she was pregnant. Caregiver responses of “Refused to answer” or “I don’t Know” were excluded. Questions regarding prenatal marijuana and alcohol exposures were also collected and defined similarly to PTE. The number of daily cigarettes smoked by the mother during the pregnancy was assessed via a follow-up question.

### Cognitive measures

The NIH Toolbox^®^ Cognitive Battery (NIHTB-CB), a validated, standardized tool for clinical research, comprised seven different tests that evaluated various cognitive processes [21]: Flanker (cognitive control and attention); List Sort Working Memory (Working Memory; Categorization; Information Processing); Dimensional Change Card Sort (Flexible thinking; concept formation; set shifting); Oral Reading Recognition (Reading decoding; Language); Pattern Comparison Processing Speed (Processing Speed; Information Processing); Picture Sequence Memory (Visuospatial sequencing & memory); and Picture Vocabulary (Language; Verbal intellect). In addition, three composite scores were generated from the seven tasks: Total Score Composite (overall cognition score derived from all tests); Fluid Intelligence Composite (included scores from Picture Sequence Memory, Dimensional Change Card Sort, Flanker Inhibitory Control and Attention, List Sorting Working Memory, and Pattern Comparison Processing Speed scores), and Crystalized Intelligence Composite (derived from Oral Reading Recognition and Picture Vocabulary scores). We examined cognitive performance across all specific tests and composite measures using uncorrected standard scores from the NIHTB-CB.

### Imaging acquisition and processing

All ABCD sites collected T1-weighted structural brain images based on a unified scan protocol, using 3T MR scanners (Siemens Prisma^®^, General Electric Discovery™ MR750 or Philips Achieva) [22, 23]. Cortical surface reconstruction and subcortical segmentation were performed by the ABCD Data Analysis, Informatics and Resource Center using multiple atlases and FreeSurfer version 5.3.0 [22]. T1-weighted data used for our study were processed with the Desikan-Killiany atlas [23] to evaluate cortical thickness and surface area of the whole brain and of selected regions of interest (ROIs), which included 21 cortical regions, and volumes of 7 selected subcortical regions from each hemisphere (Figures 2A–3A–3A). The brain regions were identified *a priori* based on previous brain MRI studies of children with PTE [11, 15, 24, 25]. To minimize the number of variables, cortical grey matter volumes were not evaluated since they are approximately the products of cortical area and thickness.

### Covariates

The covariates included age, race and ethnicity, sex, prenatal alcohol and marijuana exposures, parental education, and total annual household income, as reported by the participating parent- or caregiver in the Parent Demographic Questionnaire [26]. Parental education was a 4-level nominal variable that included: “Less than a high school diploma”, “High school, general education development (GED), or with some college but no degree”, “Associates or bachelor’s degree” and “Graduate school or higher”. Total annual household income was classified as low (<$50,000), middle ($50,000–$100,000), and high (>$100,000) by combining the categories specified in the American Community Survey [27]. For morphometric measures, hemisphere and intercranial volume (for subcortical volumes only) were additionally included as covariates.

### Statistical analysis

Statistical analyses were performed using R version 4.0.3 [28]. Demographic variables were analyzed using analysis of variance (ANOVA) or unpaired Student’s t-tests for continuous variables, and Cochran–Mantel–Haenszel tests for categorical variables. When analyzing cognitive scores and morphometric measures, we first fitted base Linear Mixed Models (LMMs) to compare children with and without PTE regardless of sex. These base models included PTE status as the fixed effect, recruitment sites (for cognitive measures) or scanner IDs (for morphometry) as random factors, with sex used as a covariate. Next, we fitted another set of base models using PTE status, sex, and the PTE-by-sex interaction as fixed effects, recruitment sites or scanner IDs as random factors and without covariates.

We also fitted covariate-adjusted LMMs which included PTE, sex, and the PTE-by-sex interaction as fixed effects, recruitment sites or scanner IDs as random effects, and hemisphere, age, prenatal alcohol and marijuana exposures, parental education, and total annual household income as covariates. We then compared the Cohen’s *d* effect size between base- and covariate-adjusted models. A significant model difference, supporting the potential impact of confounders, was identified as a difference in effect size of 15 % or more between the base- and covariate-models [29]. All LMMs and Cohen’s *d* effect size estimates were generated using the lme4 [28] and emmeans (version 1.8.7) packages [30]. All analyses employed false-discovery rate (FDR) to correct for multiple comparisons, with significance at corrected-p<0.05.

Lastly, we used the mediation package [31] to examine whether PTE-related cognitive differences were mediated by PTE-related morphometric differences. For mediation analyses, we used PTE as fixed effect, recruitment sites as random effect, and covaried for sex, age, prenatal alcohol and marijuana exposures, parental education, and total annual household income. We used 1,000 simulations to generate confidence intervals using the quasi-Bayesian Monte Carlo approach. Lastly, visualizations of brain ROIs were generated using the ggseg/ggseg3d packages [32].

## Results

### Participant characteristics

Of the ABCD baseline dataset (n=11,875), 11,609 children had complete information on PTE. The final sample included 320 (2.7 %) boys and 300 (2.6 %) girls with PTE, whereas 5,745 (49.5 %) boys and 5,244 (45.2 %) girls were unexposed (Table 1). No sex-specific effects were observed in any participant characteristics. Specifically, boys and girls with PTE did not differ significantly in age, family income, parental education, race and ethnicity, or prenatal exposures to alcohol or marijuana. The boys and girls with PTE also had similar exposure to the number of daily cigarettes smoked by the mother during the pregnancy.

**Table 1:** Participant’s characteristics by PTE status and sex (n=11,609).

Variable	Boys		Girls		p-Value
	Unexposed (n=5,745)	PTE (n=320)	Unexposed (n=5,244)	PTE (n=300)	
**Age in months, mean (SD)**	119.1 (7.50)	119.2 (7.60)	118.8 (7.47)	119.2 (7.61)	0.11^g^
**Youth’s race, n (%)** ^**a**^					
Asians	111 (1.9 %)	1 (0.3 %)	108 (2.1 %)	1 (0.3 %)	0.85^h^
Black or African American	771 (13.4 %)	74 (23.1 %)	777 (14.8 %)	72 (24 %)	
Caucasians	3,099 (53.9 %)	148 (46.3 %)	2,709 (51.6 %)	156 (52 %)	
Hispanics	1,178 (20.5 %)	46 (14.4 %)	1,113 (21.2 %)	28 (9.3 %)	
Multiracial	576 (10 %)	51 (15.9 %)	533 (10.2 %)	43 (14.3 %)	
**Family income, n (%)** ^**b**^					
<$50 K	1,449 (25.2 %)	167 (52.2 %)	1,367 (26.1 %)	168 (56.0 %)	0.69^h^
$50 K to <$100 K	1,489 (25.9 %)	88 (27.5 %)	1,365 (26.0 %)	58 (19.3 %)	
>=$100 K	2,310 (40.2 %)	32 (10.0 %)	2,094 (39.9 %)	32 (10.7 %)	
**Parental education, n (%)** ^**c**^					
<High school	335 (5.8)	36 (11.3)	353 (6.7)	44 (14.7)	0.72^h^
High school, GED or some college	1,498 (26.1)	172 (53.8)	1,331 (25.4)	137 (45.7)	
Associates or bachelor	2,414 (42.1)	96 (30.0)	2,168 (41.4)	103 (34.3)	
Graduate degree or higher	1,487 (25.9)	16 (5.0)	1,388 (26.5)	16 (5.3)	
**Parental alcohol exposure, n (%)** ^**d**^					
Unexposed	5,625 (97.9 %)	265 (82.8 %)	5,121 (97.7 %)	241 (80.3 %)	0.98^b^
Exposed	104 (1.8 %)	40 (12.5 %)	114 (2.2 %)	42 (14.0 %)	
**Prenatal alcohol exposure, n (%)** ^**e**^					
Unexposed	5,692 (99.1 %)	248 (77.5 %)	5,170 (98.6 %)	222 (74.0 %)	0.60^h^
Exposed	47 (0.8 %)	56 (17.5 %)	69 (1.3 %)	59 (19.7 %)	
**Maternal cigarettes smoked/day during pregnancy** ^**f**^					
Mean (SD)	–	7.39 (5.990)	–	7.56 (5.646)	0.22^i^

Participant characteristics are presented as mean (standard deviation, SD) or count (column %). For categorical variables, the frequency distribution between boys and girls with PTE, compared to their unexposed counterparts, were evaluated using the Cochran–Mantel–Haenszel (CMH) tests. For statistical purposes, the Multiracial and Asian racial groups were combined to generate a four-level nominal variable for the youth’s race in all our analyses. PTE, prenatal tobacco exposure; GED, a degree documenting the passing of the test of General Educational (equivalent to a high school degree). ^a^Data were missing for 15 participants (none were PTE children). ^b^Data were missing for 990 participants (75 were PTE children). ^c^Data were missing for 15 participants (none were PTE children). ^d^Data were missing for 57 participants (32 were PTE children). ^e^Data were missing for 46 participants (35 were PTE children). ^f^Data were missing for 111 participants (all PTE children). ^g^p-values derived using ANOVA. ^h^p-values derived using CMH tests. ^i^p-values derived using unpaired t-tests.

### Neurocognitive measures

**Figure 1:**
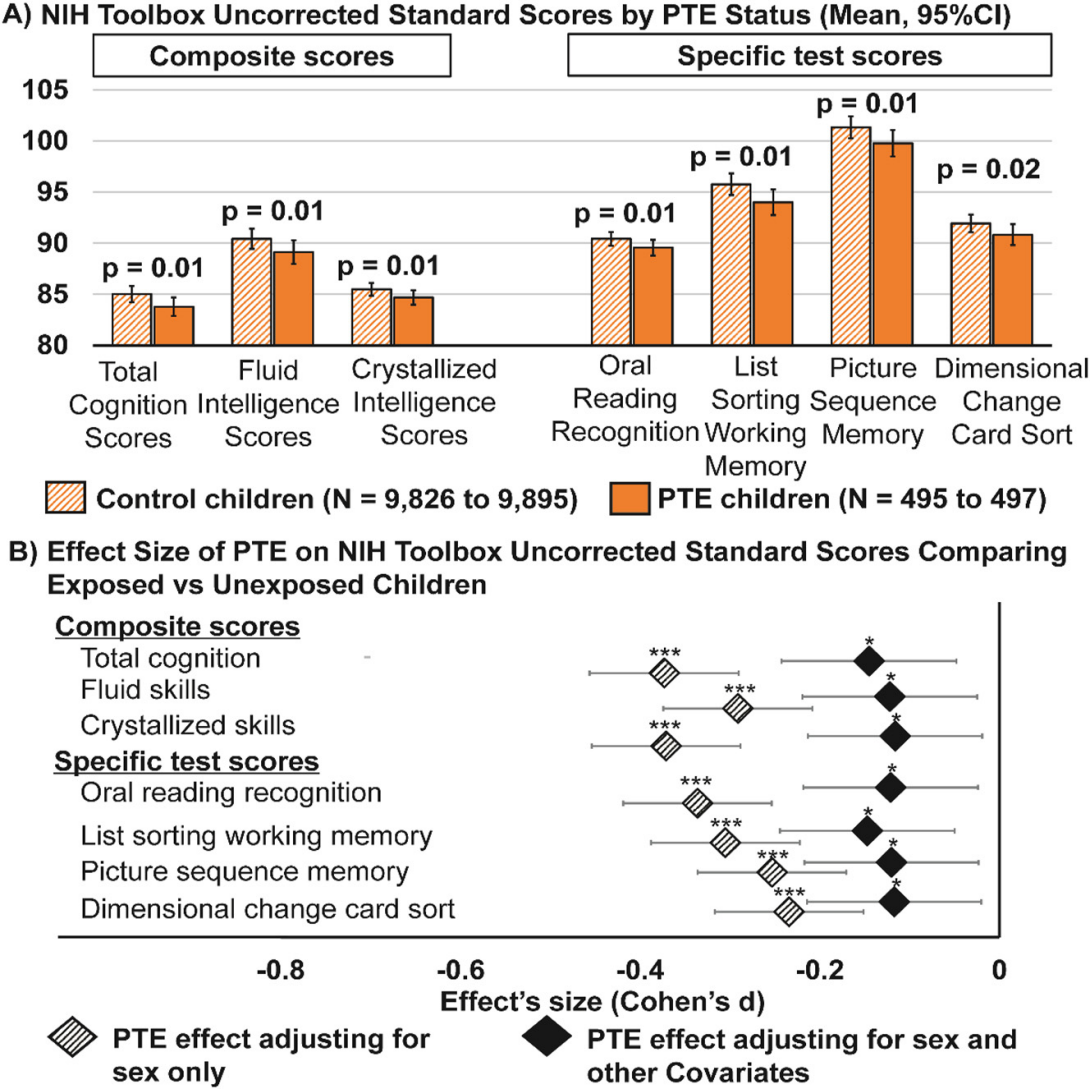
Children with PTE has poorer cognitive performance on the NIH toolbox tasks. (A) Shows estimated marginal means and 95 % confidence intervals (error bars) by PTE status of neurocognitive measures that showed significant PTE effect after adjustment for covariates, calculated using linear mixed effects models (LMMs) (p-values are from LMMs corrected for multiple comparison using false discovery rate); (B) plots the effect size (Cohen’s *d*) and 95 % confidence intervals of PTE on cognitive scores first adjusting for sex only (base model) and then for sex and other covariates (covariate-adjusted model). The covariate-adjusted model included: the youth’s sex, age and race, parental education, annual family income, prenatal exposures to marijuana and alcohol as fixed effects, and ABCD site ID as random effect. Negative effect sizes indicate a lower cognitive score when comparing children with PTE to controls. Asterisks used in (B) are for the significance of the group differences between children with PTE versus controls before and after adjustment for covariates in the LMMs, where: ^*^p-value <0.05, ^**^p-value <0.01 and ^***^p-value <0.001.

Children with PTE had lower scores than unexposed children in all three composite scores and in 4 out of 7 specific test scores (Figure 1A, Supplementary Table 1). However, we did not find sex-specific PTE effects on neurocognitive measures. Thus, both the base- and covariate-adjusted models did not include the PTE-by-sex interaction terms in the analyses for cognitive scores.

**Figure 2:**
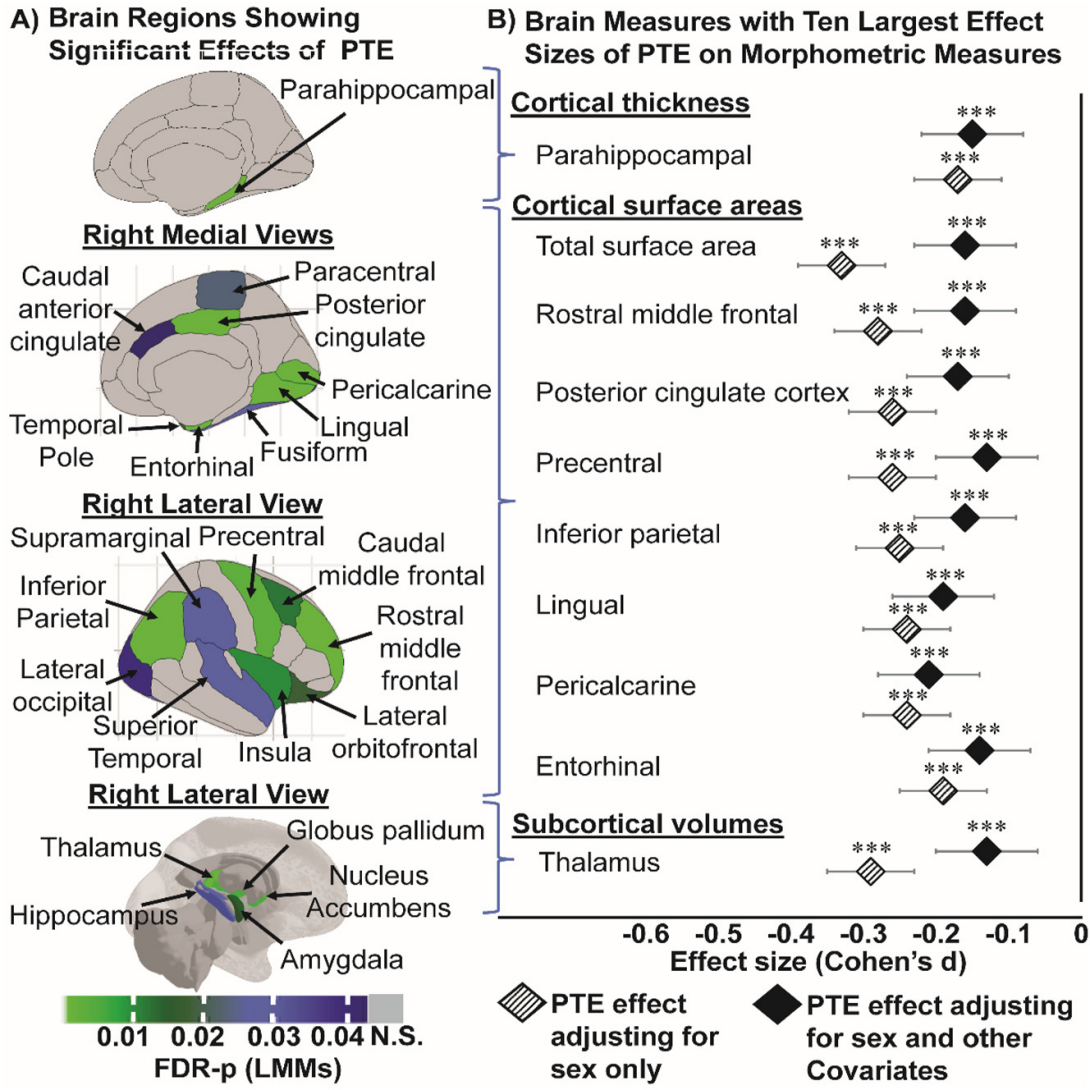
Children with PTE has thinner cortices (in mm), smaller surface areas (in mm^2^) and smaller subcortical volumes (in mm^3^). (A) Shows all the region-specific brain measures that showed a significant PTE effect after adjusting for covariates, calculated using linear mixed models (FDR-p: p-values corrected for multiple comparison using false discovery rate). Top image: right hemisphere medial view of region(s) that showed significant PTE effects on cortical thickness; middle images (top & bottom): right hemisphere medial and lateral views of region(s) that showed significant PTE effects on surface area, respectively; and bottom image: right hemisphere lateral view of region(s) that showed significant PTE effects on subcortical volumes; (B) plots the top 10 effect sizes (Cohen’s *d*) and 95 % confidence intervals calculations for regional morphometric measures in (A) that showed the strongest associations with PTE first adjusting for sex only (base model) and then for sex and other covariates (covariate-adjusted model). The covariate-adjusted model included: the youth’s sex, age and race, parental education, annual family income, prenatal exposures to marijuana and alcohol as fixed effects, and MRI scanner ID as random effect. Negative effect sizes indicate a thinner cortex, or smaller areas or subcortical volumes in children with PTE compared to unexposed controls. Asterisks used in (B) are for the significance of group differences between children with and without PTE before and after adjustment for covariates in the LMMs, where: ^*^p-value <0.05, ^**^p-value <0.01 and ^***^p-value <0.001.

**Figure 3:**
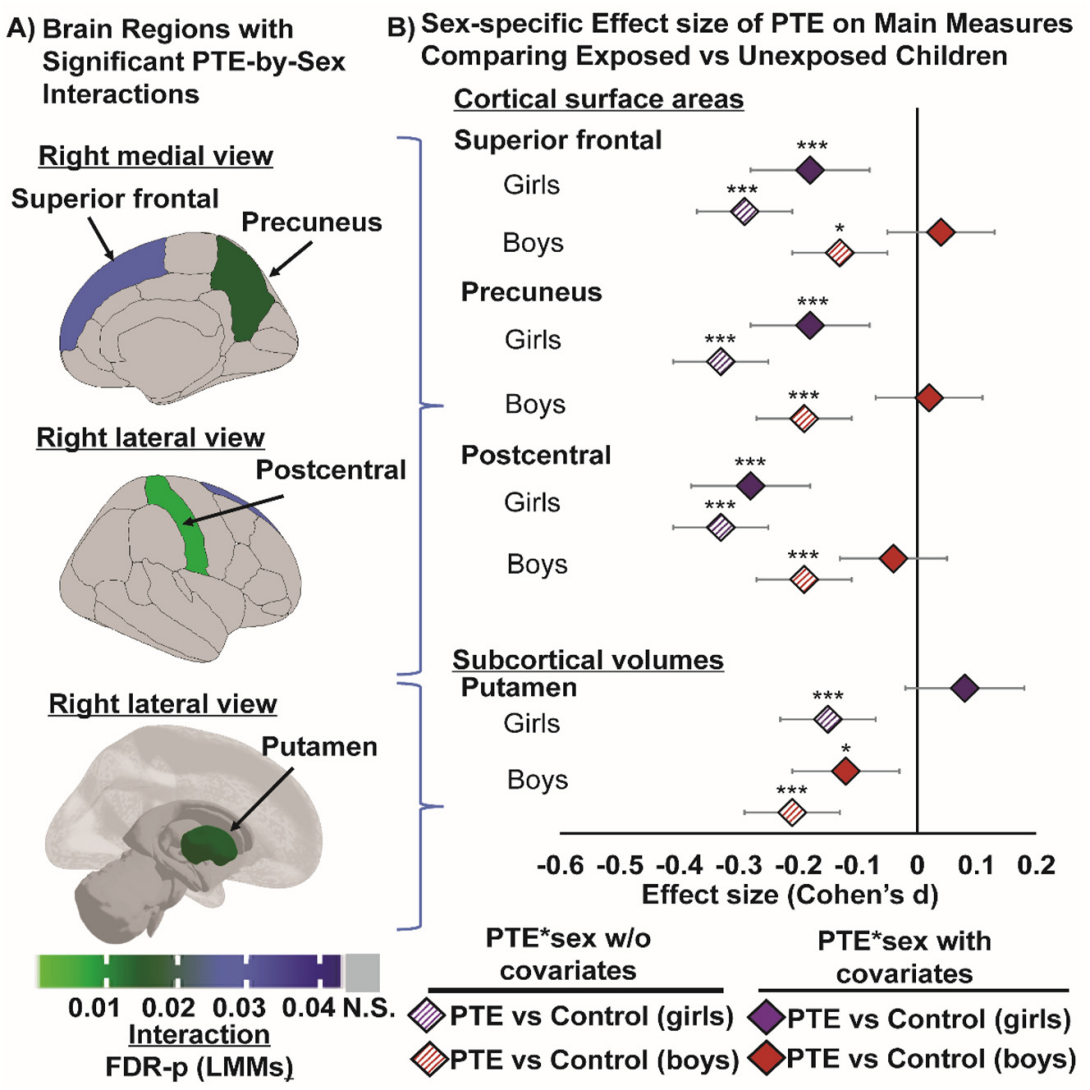
Sex-specific PTE effects are observed in cortical surface areas (in mm^2^) and subcortical volumes (in mm^3^). (A) Shows region specific brain measures that showed a significant sex-specific PTE effect (linear mixed models, adjusted for covariates, p-values are corrected using FDR). Top & middle images: show right medial and lateral views of region(s) that showed a significant sex-by-PTE interaction in our analyses of cortical surface areas; bottom image: shows right hemisphere lateral view of region(s) that showed a significant sex-PTE interaction in our analyses of subcortical volumes; (B) plots the Cohen’s *d* effect size and 95 % confidence intervals of sex-specific PTE effects on cortical surface area (mm^2^) and subcortical volume (mm^3^), and for each region shown in (A) first adjusting for sex only (base model without other co-variates) and then for sex and other covariates (covariate-adjusted model). The covariate-adjusted model included: the youth’s sex, age and race, parental education, annual family income, prenatal exposures to marijuana and alcohol as fixed effects, and MRI scanner ID as random effect. Negative values of effect sizes indicate a smaller surface area or subcortical volume when comparing each subgroup of children with PTE to their control counterparts. Asterisks in (B) indicate significant group differences between children with PTE versus controls before and after adjustment for covariates from *post hoc* analyses, where: ^*^p-value <0.05, ^**^p-value <0.01 and ^***^p-value <0.001.

The greatest PTE differences were observed for the total and crystallized intelligence composites (Cohen’s *d:* −0.37; 95 % CI: −0.45 to −0.29), followed by reading decoding (Cohen’s *d*: −0.34; 95 % CI: −0.42 to −0.26) from the base model. PTE main effects remained significant after covariate adjustments, although all effect sizes became smaller (Figure 1B).

### Brain morphometry

#### Cortical thickness

Children with PTE had thinner parahippocampal gyri (Cohen’s *d*: −0.15; 95 % CI: −0.23 to −0.11, Figure 2A and B, Supplementary Table 2) than unexposed children; the effect sizes were similar between the base- and covariate-adjusted models (Figure 2B, Supplementary Table 3). No PTE main effect or sex-specific PTE effects on the other cortical thickness measures were observed (data not shown).

#### Cortical surface area

Children with PTE had smaller cortical surface areas than unexposed children for total cortical surface area (Cohen’s *d*: −0.16; 95 % CI: −0.23 to −0.09), and in several regions in the frontal, parietal and temporal lobes (Figure 2A, Supplementary Table 2). Except for the pericalcarine cortical area, the PTE effect sizes across surface areas were attenuated but remained significant after covariate adjustments (Figure 2B, Supplementary Table 3).

PTE-by-sex interactions were found in the covariate-adjusted models for surface areas in the superior frontal (interaction-FDR-p=0.01), precuneus (interaction-FDR-p=0.03), and postcentral gyri (interaction-FDR-p=0.02) (Figure 3A, Supplementary Table 2). Specifically, after adjusting for covariates, only exposed girls had smaller surface areas than their unexposed counterparts in the superior frontal [(Cohen’s *d*: −0.18; 95 % CI: −0.28 to – 0.08), postcentral (Cohen’s *d*: −0.28; 95 % CI: −0.38 to −0.18), and precuneus (Cohen’s *d*: −0.18; 95 % CI: −0.28 to –0.08); Figure 3B). Except for the postcentral cortical area, the sex-specific PTE effect sizes were attenuated but remained significant after covariate adjustments (Figure 3B).

#### Subcortical volumes

Children with PTE had smaller thalamic, amygdalar, hippocampal, pallidal and nucleus accumbens but similar caudate volumes than unexposed children (Figure 2A, Supplementary Table 2). The greatest PTE effect was observed for the thalamic volume from the base model (Cohen’s *d*: −0.29; 95 % CI: −0.35 to −0.23). PTE effect sizes across subcortical volumes were attenuated but remained significant after covariate adjustments (Figure 2B, Supplementary Table 3). A PTE-by-sex interaction in the putamen was significant from covariate-adjusted models (interaction-FDR-p=0.02) (Figure 3A, Supplementary Table 2). Only exposed boys, but not girls, showed smaller putamen volume than their unexposed counterparts (Cohen’s *d*: −0.12; 95 % CI: −0.21 to −0.03; Figure 3B, Supplementary Table 4).

In addition, the number of daily cigarettes smoked by the mothers during pregnancy did not correlate with the cortical thickness, surface areas or subcortical volumes in children with PTE (data not shown).

#### Mediation analysis

**Figure 4:**
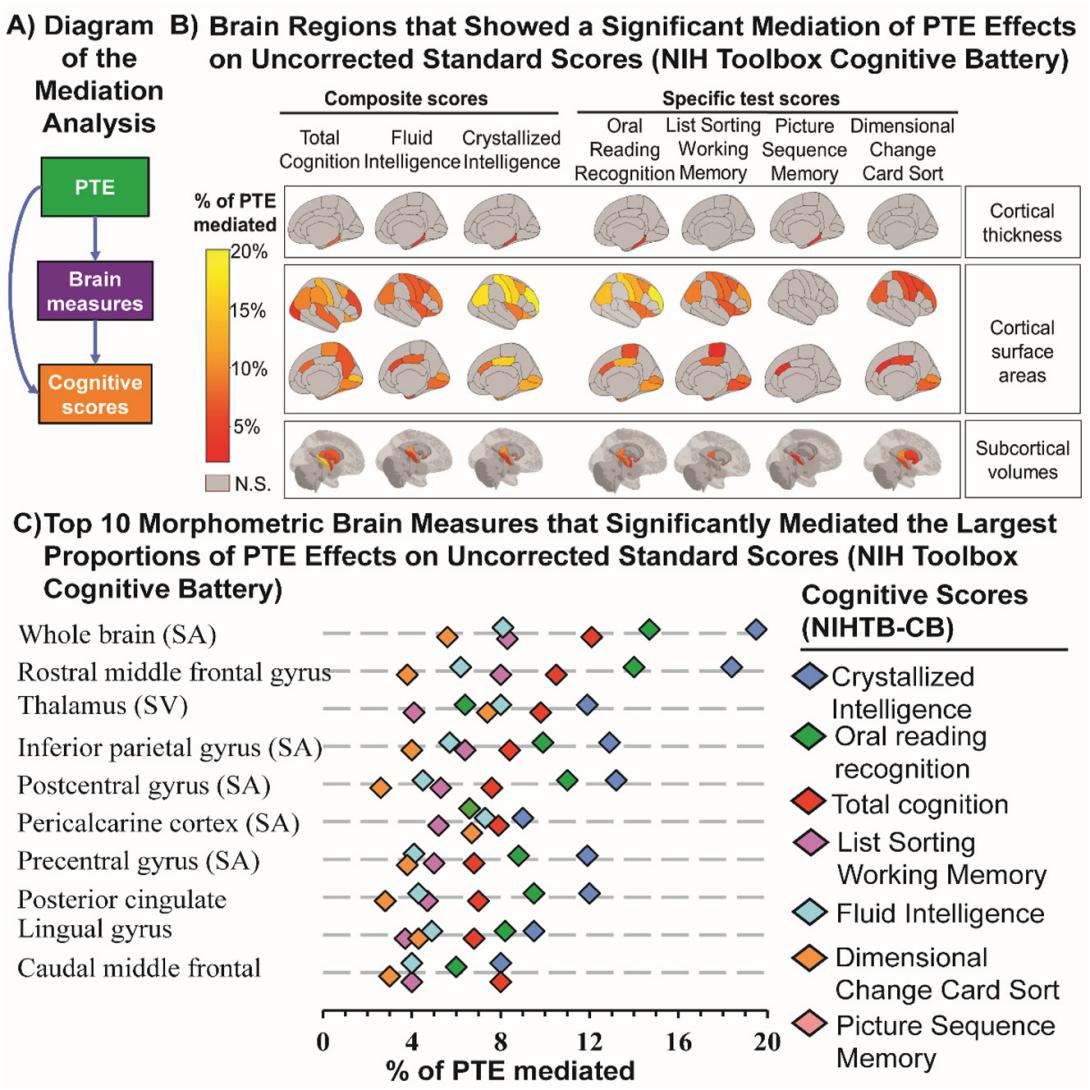
Poorer cognitive scores in the NIH toolbox are associated with PTE effects that were mediated by cortical thickness, surface area and subcortical volumes. (A) Shows a general outline for the mediation analyses that evaluated the proportion of the PTE effect on cognitive measures that was partially mediated by morphometric brain measures. (B) Shows regional mediated effects for each cognitive score in the NIH toolbox that was associated with PTE, presented as percentage of the total PTE effect mediated by morphometric measure (% PTE mediated). All mediation analyses covaried for the youth’s sex, age and race, the parental education, annual family income, prenatal exposures to marijuana and alcohol, hemisphere, and intercranial volume (subcortical volumes) as fixed effects, and ABCD site ID as random effect. We used the false discovery rate approach to correct for multiple comparisons, with significance set at p<0.05. (C) Shows the top 10 morphometric brain measures the explained the largest proportions of the PTE effects on cognitive scores. S.A. = surface area; S.V. = subcortical volume.

Cortical surface areas and subcortical brain volumes mediated the relationships between PTE and cognitive measures (Figure 4A and B, Supplementary Tables 5A–B). The 10 strongest mediation effects were observed for the total cortical surface area, the regional surface areas of the frontal and parietal lobes, and thalamic volume (Figure 4B and C). For example, exposed children had poorer crystallized composite scores and 19.5 % (95 % CI: 7.8–61.1 %, p=0.019) of that difference was mediated by their smaller total cortical surface area compared to unexposed children (Figure 4B and C, Supplementary Table 5A).

## Discussion

Compared to unexposed children, children with PTE in the ABCD study had poorer cognitive performance in reading decoding, executive and memory functions, which led to the lower composite scores on overall cognition and crystallized and fluid intelligence. Children with PTE also had thinner parahippocampal gyri, smaller total cortical surface area and in regions of the frontal, parietal, and temporal lobes, as well as smaller thalamic and basal ganglia volumes than unexposed children. Additionally, PTE main effects remained significant but with smaller effect sizes after adjusting for age, sex, race and ethnicity, family income, hemisphere and prenatal alcohol and marijuana exposures. Furthermore, we found sex-specific PTE effects in the cortical surface area of the precuneus, superior frontal and postcentral gyri and in putamen volumes. Finally, we showed that the associations between PTE and poor cognitive scores were partially mediated by smaller total surface areas and areas across frontal and parietal regions, as well as smaller thalamic volumes.

## PTE is associated with poor neurocognitive function

We found children with PTE had relatively poorer overall cognition, cognitive flexibility and working memory. These results are consistent with prior studies that also found poorer overall cognition and executive function (a process that requires working memory, cognitive flexibility, and self-control) among children with PTE [6, 33]. In addition, children with PTE in this study also had poorer reading decoding, which is associated with decoding, executive function, and memory from previous studies [34, 35]. For example, children with poor executive function had reading comprehension problems, both directly and indirectly, through poor decoding ability [36]. In addition, compared to unexposed controls, 7–9 years old children with PTE had poorer reading, specifically decoding, which was attributed to their poorer memory skills [35].

In contrast, prior studies with smaller sample size have reported PTE had no negative impact on cognitive measures in children after adjustments for socioeconomic status and other demographics [37, 38]. Our current larger study confirmed that children with PTE had poorer cognitive performance, although the association between PTE and cognition was partially confounded by the co-occurrence of other prenatal drug exposures and sociodemographic factors.

## PTE is associated with thinner parahippocampal cortical thickness

Children with PTE had thinner parahippocampal gyri even after adjustment for covariates. This finding is consistent with prior studies that showed adolescents with PTE had thinner cortices in frontal, parietal and temporal brain regions compared to unexposed adolescents [15]. However, another study showed that younger children with PTE (6–8-year-olds) also had thinner cortices in the superior frontal, superior parietal, and precentral cortices, but not in the parahippocampal gyrus [11]. These study discrepancies may be due to that cortical brain development at variable rate across different brain regions [39]. Hence, it would be important to follow our participants to assess how PTE may influence the developmental trajectories of specific cortical regions, especially throughout puberty. The current study establishes a pre-pubertal starting point for these exposed children.

## PTE and PET-sex interaction in smaller cortical surface areas

Children with PTE had smaller total cortical surface areas and smaller regional surface areas in the frontal, temporal, and parietal lobes, compared to unexposed children. These findings are consistent with a recent study that also reported smaller surface areas across similar brain regions among 9-11-year-old children with PTE [25]. Multiple animal studies demonstrated several mechanisms by which PTE or nicotine affects brain development [7, 40]. Exposure to nicotine disrupted neuronal migration [7], which may alter cortical thickness by disrupting radial migration, and influence surface area development by disrupting tangential dispersion of migrating neurons in the developing cortex [41]. These mechanisms could explain the smaller surface areas and thinner cortical gray matter in our exposed children.

Cortical development is also influenced by sex hormones. In typically developing adolescents, higher testosterone levels were associated with smaller superior frontal surface areas in girls, but not in boys [42]. Furthermore, animal studies showed that PTE was associated with higher testosterone levels in exposed fetuses and adolescent offspring [43] as compared to unexposed. Similarly, higher testosterone levels were observed in the PTE-exposed human offspring as adolescents and young adults relative to unexposed offspring [44]. Therefore, PTE might lead to higher levels of testosterone, which might result in smaller surface areas on the superior frontal, postcentral and precuneus gyri in our girls with PTE, but not in the boys, compared to their unexposed counterparts.

## PTE and PTE-sex interaction in smaller subcortical volumes

Consistent with previous studies and regardless of sex, children with PTE had smaller thalamic and amygdalar volumes [12, 13, 15, 45]. However, our study showed that only PTE-boys had smaller putamen than the unexposed boys. PTE is related to earlier onset of pubertal development [46], which was associated with sex-specific changes in the subcortical brain volumes. For example, boys (7–20 years) with greater pubertal maturity had smaller putamen volumes than their age-matched peers [47]. Thus, the smaller putamen volume in PTE-boys in our study might be due to earlier onsets of puberty. However, it is worth noting that the associations between pubertal development, sex hormones and brain maturation vary widely between brain regions, morphometric measures, and sex [42, 46]. Future studies should evaluate the relationships between PTE, pubertal sex hormones and brain development.

## Mediation analyses

This is the first study that clearly demonstrate that the lower performance on executive control and reading decoding in children with PTE are partially attributed to their delayed brain development in thalamus and frontoparietal cortical surface area. Our findings are consistent with and validated prior studies that found children with PTE had poorer cognitive scores globally and in specific domains, including reading skills, executive and memory functions [6, 33, 48], as well as smaller thalamic volumes and thinner frontal, parietal and precentral cortices [11, 45].

Similar altered brain development pattern has been found in children with attention deficit hyperactivity disorder (ADHD) previously, that children with ADHD had smaller prefrontal cortical area and thalamic volumes compared to typically developing children [49]. Since the development of cortical surface area is associated with formation of thalamo-cortical connections, thalamic structural abnormalities may be linked to altered cortical surface areas [49]. Other studies have also shown that portions of the cortico-striatal-thalamo-cortical circuits, which subserve executive functions, are vulnerable to prenatal tobacco exposure [45, 50]. Therefore, poorer executive control processes in our children with PTE might be related to alterations of these circuits involved in executive function. Our findings are also consistent with prior studies that found children with PTE had more parent-reported attention problems [45], and altered or inefficient activation of the working-memory network during fMRI [50].

## Strengths and limitations

The present study has several strengths. First, our large sample is derived from a nationally and demographically diverse study population. Second, we included important covariates that often co-occur with PTE, such as factors associated with lower socioeconomic status and exposures to prenatal alcohol and marijuana. Lastly, since the ABCD study is longitudinal, future studies can follow these youth to determine how brain developmental trajectories and cognitive maturation may be influenced by PTE, and how this might interact with initiation of tobacco use, or increase risk for tobacco use [14], which could lead to additive effects on brain and cognitive deficits [9].

This study also has several limitations. First, information on PTE was obtained from parent self-reports. Self-reporting might have introduced some misclassification due to underreporting of prenatal tobacco use, which could have led to underestimation of the effects observed in this study. Second, we did not evaluate the risk for postnatal passive smoke exposure which might also affect cognitive and brain development in children. Furthermore, because we only evaluated the baseline data, this cross-sectional study limits our ability to infer causality on our findings related to PTE. Thus, follow-up data on cognitive and morphometric brain measures are needed and will become available as the ABCD study continues to collect data from this cohort. Lastly, we only found minimal sex-specific effects of PTE on brain surface area, which may be because these children were recruited primarily at pre-pubertal stage. Future longitudinal studies should evaluate sex-specific PTE effects on cognitive and brain changes during pubertal development, including the pubertal hormone levels.

## Conclusions

Our findings suggest that PTE is associated with abnormal brain morphometry, which in turn contribute to their relatively poorer cognitive performance in preadolescents. Our findings expand the existing literature by demonstrating that PTE is associated with smaller cortical surface areas in children and that such effects may be sex-specific.

## Supplementary Material

Supplementary Material Details

Supplementary Material Details

Supplementary Material Details
