# Supplementary material for: Prenatal tobacco exposure on brain morphometry partially mediated poor cognitive performance in preadolescent children
Source: NeuroImmune Pharm Ther. 2023 Jul 13;2(4):375–86. doi: 10.1515/nipt-2023-0013 (PMC10696570; doi:10.1515/nipt-2023-0013)
Supplement: Supplementary file 1 — Supplementary Material Details [file j_nipt-2023-0013_suppl_001.pdf]

Supplemental Table 1. The effect of prenatal tobacco exposure (PTE) on cognitive measures (uncorrected standard scores in the NIH Toolbox) before and after adjustment for covariates.

		Mean differences ($Mean_{diff}$): PTE vs. Non-PTE Children										
		**Base model[a]**					**Covariate-adjusted model[b]**					
		**PTE**	**Non-PTE**		**LMM-*p*[c]**		**PTE**	**Non-PTE**		**LMM-p[c]**		
**Scores**	**Measure**	**(N)**	**(N)**	**M_{diff} (S.E.)**	PTE	**Sex**	**(N)**	**(N)**	**$Mean_{diff}$ (S.E.)**	PTE	**Sex**	PTE*Sex
**Composite scores**												
Total composite scores	Overall Cognition	609	10,765	-3.29 (0.37)	<0.001	<0.001	495	9,826	-1.25 (0.37)	0.01	< 0.001	-
Crystallized composite scores	Crystallized Intelligence	612	10,820	-2.55 (0.29)	<0.001	0.01	497	9,872	-0.80 (0.29)	0.01	0.38	-
Fluid composite scores	Fluid Intelligence	609	10,769	-3.0 (0.44)	<0.001	<0.001	495	9,830	-1.30 (0.47)	0.01	< 0.001	-
**Specific tests**												
Oral reading recognition	Reading Decoding	612	10,838	-2.29 (0.29)	<0.001	0.95	497	9,888	-0.86 (0.30)	0.01	0.61	-
List sorting working memory	Working Memory	609	10,807	-3.62 (0.50)	<0.001	0.001	495	9,863	-1.76 (0.55)	0.01	0.002	-
Picture sequence memory	Episodic Memory	612	10,840	-3.02 (0.50)	<0.001	<0.001	497	9,889	-1.55 (0.57)	0.01	< 0.001	-
Dimensional change card sort	Cognitive Flexibility	612	10,847	-2.18 (0.39)	<0.001	<0.001	497	9,895	-1.1 (0.44)	0.02	< 0.001	-

Supplemental Table 1. Shows the estimated marginal mean differences in cognitive scores from the NIH Toolbox between children with and without PTE first adjusting for sex only (Base model) and then for sex and other covariates (Covariate-adjusted model), which were derived using linear mixed models (LMM). Mean differences are presented along with their standard errors in parentheses. We used the uncorrected scores in the NIH Toolbox for this analysis.

[a]Fixed effects in the base model included PTE status and child's sex

[b]Fixed effects in the covariate-adjusted model included PTE status, child's age, sex and race/ethnicity, the PTE*sex interaction, parental education, average annual family income, and prenatal alcohol and marijuana exposures.

[a,b]We included ABCD site IDs as random effects in both models.

[c]*p*-values derived from linear mixed effect models (LMM) were corrected for multiple comparison using the false discovery rate approach

Dashes indicate that the PTE*sex interaction term did not survive FDR corrections and were removed from the model.

Supplemental Table 2. Brain regions showing significant PTE effect on cortical thickness (mm), surface area (mm^2) and subcortical volume (mm^3) before and after adjustment for covariates.

		Mean differences (Mean$_{diff}$): PTE vs. Non-PTE Children										
		Base model[a]					Covariate-adjusted model[b]					
		PTE	Non-PTE	Meandiff	LMM-p[c]		PTE	Non-PTE	Meandiff	LMM-p[c]		
**Brain measues**	**Location**	**(N)**	**(N)**	**(S.E.)**	PTE	**Sex**	**(N)**	**(N)**	**(S.E.)**	PTE	**Sex**	PTE*Sex
**Cortical thickness (mm)**												
Parahippocampal gyrus	Temporal	608	10,861	-0.04 (0.01)	<0.001	<0.001	496	9,913	-0.04 (0.01)	<0.001	<0.001	-
**Cortical surface areas (mm^2)**												
Total cortical surface area	Global	608	10,861	-2,508 (231)	<0.001	<0.001	496	9913	-1,197 (257)	<0.001	<0.001	-
Caudal anterior cingulate	Frontal	608	10,861	-27 (5)	<0.001	<0.001	496	9,913	-15 (6)	0.01	<0.001	-
Caudal middle frontal	Frontal	608	10,861	-84 (14)	<0.001	<0.001	496	9,913	-46 (15)	<0.001	<0.001	-
Lateral orbitofrontal	Frontal	608	10,861	-61 (9)	<0.001	<0.001	496	9,913	-30 (10)	0.01	<0.001	-
Paracentral	Frontal	608	10,861	-30 (7)	0.01	<0.001	496	9,913	-22 (8)	0.01	<0.001	-
Precentral	Frontal	608	10,861	-150 (17)	<0.001	<0.001	496	9,913	-71 (20)	<0.001	<0.001	-
Rostral middle frontal	Frontal	608	10,861	-222 (24)	<0.001	<0.001	496	9,913	-124 (27)	<0.001	<0.001	-
Superior frontal	Frontal	608	10,861	-181 (26)	<0.001	<0.001	496	9,913	-67 (30)	0.65	<0.001	0.01
Inferior parietal	Parietal	608	10,861	-223 (27)	<0.001	<0.001	496	9,913	-116 (25)	<0.001	<0.001	-
Postcentral	Parietal	608	10,861	-138 (16)	<0.001	<0.001	496	9,913	-85 (18)	0.68	<0.001	0.02
Posterior cingulate	Parietal	608	10,861	-55 (6)	<0.001	<0.001	496	9,913	-36 (7)	<0.001	<0.001	-
Precuneus	Parietal	608	10,861	-113 (16)	<0.001	<0.001	496	9,913	-43 (18)	0.66	<0.001	0.03
Supramarginal	Parietal	608	10,861	-135 (18)	<0.001	<0.001	496	9,913	-56 (20)	0.01	<0.001	-
Entorhinal	Temporal	608	10,861	-17 (3)	<0.001	<0.001	496	9,913	-12 (3)	<0.001	<0.001	-
Fusiform	Temporal	608	10,861	-93 (12)	<0.001	<0.001	496	9,913	-38 (14)	0.01	<0.001	-
Superior temporal	Temporal	608	10,861	-93 (13)	<0.001	<0.001	496	9,913	-40 (15)	0.01	<0.001	-
Temporal pole	Temporal	608	10,861	-9 (2)	<0.001	<0.001	496	9,913	-6 (2)	0.01	<0.001	-
Lateral occipital	Occipital	608	10,861	-109 (18)	0.003	<0.001	496	9,913	-51 (20)	0.01	<0.001	-
Lingual	Occipital	608	10,861	-99 (12)	<0.001	<0.001	496	9,913	-77 (14)	<0.001	<0.001	-
Pericalcarine	Occipital	608	10,861	-57 (7)	<0.001	<0.001	496	9,913	-50 (8)	<0.001	<0.001	-
Insula	Insular	608	10,861	-41 (7)	<0.001	<0.001	496	9,913	-26 (8)	<0.001	<0.001	-
**Subcortical volumes (mm^3)**												
Amygdala	Limbic	608	10,861	-48 (6)	<0.001	<0.001	496	9,914	-16 (6)	0.02	<0.001	-
Hippocampus	Limbic	608	10,861	-102 (12)	<0.001	<0.001	496	9,914	-27 (12)	0.03	<0.001	-
Globus pallidum	Basal ganglia	608	10,861	-52 (7)	<0.001	<0.001	496	9,914	-24 (7)	<0.001	<0.001	-
Nucleus Accumbens	Basal ganglia	608	10,861	-25 (3)	<0.001	<0.001	496	9,914	-10 (3)	<0.001	0.03	-
Putamen	Basal ganglia	608	10,861	-117 (19)	<0.001	<0.001	496	9,914	-12 (19)	0.02	<0.001	0.02
Thalamus	Diencephalon	608	10,861	-199 (21)	<0.001	<0.001	496	9,914	-60 (16)	<0.001	<0.001	-

Supplemental Table 2. Shows the mean differences in cortical thickness (in mm), cortical surface areas (in mm^2) and subcortical volumes (in mm^3) between children with and without PTE first adjusting for sex only (Base model) and then for sex and other covariates (Covariate-adjusted model), which were derived using linear mixed models (LMM). Mean differences are presented along with their standard errors in parentheses.

[a]Fixed effects in the base model included PTE status and child's sex

[b]Fixed effects in the covariate-adjusted model included PTE status, child's age, sex and race/ethnicity, hemisphere, the PTE*sex interaction, parental education, average annual family income, and prenatal alcohol and marijuana exposures. Intercranial volume was included as an additional fixed effect for volumetric analyses of subcortical structures only.

[a,b]We included scanner IDs as random effects in both models.

[c]*p*-values from linear mixed effect models (LMM) were corrected for multiple comparison using the false discovery rate approach

Dashes indicate that the PTE*sex interaction term did not survive FDR corrections and were removed from the model.

Supplemental Table 3. Effect size estimates for the association between morphometric measures and PTE status before and after adjustment of covariates.

		Cohen's *d*: Effect sizes (95% CI) PTE vs Non-PTE Children		
**Brain measues**	**Location**	**Base model[a]**	**Covariate-adjusted[b]**	%Change[c]
**Cortical thickness (mm)**				
Parahippocampal gyrus	Limbic	-0.17 (-0.23 to -0.11)	-0.15 (-0.22 to -0.08)	12
**Cortical surface areas (mm^2)**				
Total cortical surface area	Global	-0.32 (-0.38 to -0.26)	-0.16 (-0.23 to -0.09)	50
Caudal anterior cingulate	Frontal	-0.15 (-0.21 to -0.09)	-0.09 (-0.16 to -0.02)	40
Caudal middle frontal	Frontal	-0.18 (-0.24 to -0.12)	-0.11 (-0.17 to -0.05)	39
Lateral orbitofrontal	Frontal	-0.20 (-0.26 to -0.14)	-0.10 (-0.17 to -0.03)	50
Paracentral	Frontal	-0.12 (-0.18 to -0.06)	-0.10 (-0.17 to -0.03)	17
Precentral	Frontal	-0.26 (-0.31 to -0.20)	-0.13 (-0.19 to -0.05)	50
Rostral middle frontal	Frontal	-0.28 (-0.34 to -0.22)	-0.16 (-0.23 to -0.09)	43
Inferior parietal	Parietal	-0.25 (-0.31 to -0.19)	-0.16 (-0.23 to -0.09)	36
Posterior cingulate	Parietal	-0.26 (-0.32 to -0.20)	-0.17 (-0.24 to -0.10)	35
Supramarginal	Parietal	-0.22 (-0.28 to -0.16)	-0.10 (-0.17 to -0.03)	55
Entorhinal	Limbic	-0.19 (-0.25 to -0.13)	-0.14 (-0.21 to -0.07)	26
Fusiform	Temporal	-0.22 (-0.27 to -0.17)	-0.09 (-0.16 to -0.02)	59
Superior temporal	Temporal	-0.21 (-0.27 to -0.15)	-0.09 (-0.16 to -0.02)	57
Temporal pole	Temporal	-0.14 (-0.19 to -0.09)	-0.10 (-0.17 to -0.03)	29
Lateral occipital	Occipital	-0.18 (-0.24 to -0.12)	-0.09 (-0.16 to -0.02)	50
Lingual	Occipital	-0.24 (-0.30 to -0.18)	-0.19 (-0.26 to -0.12)	21
Pericalcarine	Occipital	-0.24 (-0.30 to -0.18)	-0.21 (-0.28 to -0.14)	13
Insula	Insular	-0.17 (-0.23 to -0.11)	-0.11 (-0.18 to -0.04)	35
**Subcortical volumes (mm^3)**				
Amygdala	Limbic	-0.24 (-0.30 to -0.18)	-0.09 (-0.16 to -0.02)	63
Hippocampus	Limbic	-0.25 (-0.31 to -0.19)	-0.08 (-0.15 to -0.01)	68
Globus pallidum	Basal ganglia	-0.21 (-0.27 to -0.15)	-0.12 (-0.18 to -0.06)	43
Nucleus Accumbens	Basal ganglia	-0.24 (-0.30 to -0.18)	-0.12 (-0.19 to -0.05)	50
Thalamus	Diencephalon	-0.29 (-0.35 to -0.23)	-0.13 (-0.20 to -0.06)	55

Supplemental Table 3. Compares the marginal difference in effect size (Cohen's *d*) between children with and without prenatal tobacco exposure (PTE) for all regions examined, first adjusting for sex only (Base model) and then for sex and other covariates (Covariate-adjusted model). Effect size estimates are shown for morphometric brain measures associated with PTE, and they are presented along with their 95% confidence intervals in parentheses.

[a]Fixed effects in the base model included PTE status and child's sex

[b]Fixed effects in the covariate-adjusted model included PTE status, child's age, sex and race/ethnicity, hemisphere, parental education, average annual family income, and prenatal alcohol and marijuana exposures. Intercranial volume was included for volumetric analyses of subcortical structures only.

[a,b] We included scanner IDs as random effects in both models.

[c]The change in effect size between the base and covariate-adjusted models are presented in percentages

Supplemental Table 4. Brain regions showing significant sex-specific PTE effects on surface area (mm^2) and subcortical volume (mm^3) before and after adjustment for covariates.

		Mean differences (M_{diff}): Boys with PTE vs. Boys without PTE							
		Base model[a]				Covariate-adjusted model[b]			
Brain measues	Location	PTE (N)	Non-PTE (N)	M_{diff} (S.E.)	M_{diff}-p[c]	PTE (N)	Non-PTE (N)	M_{diff} (S.E.)	M_{diff}-p[c]
**Cortical surface areas (mm^2)**									
Superior frontal[c]	Frontal	316	5,698	-114 (36)	0.01	264	5176	25 (39)	0.92
Postcentral[c]	Parietal	316	5,698	-101 (22)	< 0.001	264	5176	-25 (24)	0.72
Precuneus[c]	Parietal	316	5,698	-80 (22)	< 0.001	264	5176	8 (23)	0.99
**Subcortical volumes (mm^3)**									
Putamen	Basal ganglia	316	5,698	-136 (26)	< 0.001	264	5176	-66 (25)	0.01

		Mean differences (Mdiff): Girls with PTE vs. Girls without PTE							
		Base model[a]				Covariate-adjusted model[b]			
Brain measues	Location	PTE (N)	Non-PTE (N)	M_{diff} (S.E.)	M_{diff}-p[c]	PTE (N)	Non-PTE (N)	M_{diff} (S.E.)	M_{diff}-p[c]
**Cortical surface areas (mm^2)**									
Superior frontal[c]	Frontal	292	5,163	-254 (38)	< 0.001	232	4737	-157 (42)	< 0.001
Postcentral[c]	Parietal	292	5,163	-178 (23)	< 0.001	232	4737	-145 (25)	< 0.001
Precuneus[c]	Parietal	292	5,163	-150 (23)	< 0.001	232	4737	-93 (25)	< 0.001
**Subcortical volumes (mm^3)**									
Putamen	Basal ganglia	292	5,163	-98 (27)	< 0.001	232	4737	42 (27)	0.13

Supplemental Table 4. Shows the estimated marginal mean differences in cortical surface areas (in mm^2) and subcortical volumes (in mm^3) between children with and without PTE stratified by sex. Mean differences are shown before and after adjustment for covariates, which were derived using Linear Mixed Models (LMMs) and pairwise comparisons. Mean differences (M_{diff}) are presented along with their standard errors in parentheses.

[a]Fixed effects in the base model included PTE status and child's sex

[b]Fixed effects in the covariate-adjusted model included PTE status, child's age, sex and race/ethnicity, hemisphere, the PTE status-by-sex interaction, parental education, average annual family income, and prenatal alcohol and marijuana exposures. Intercranial volume was included for the analyses of putamen volume only.

[a,b]We included scanner IDs as random effects in both models.

[c]*p*-values for the pairwise comparison, which were corrected using the Tukey method

d*p*-values from linear mixed effect models (LMMs) were corrected for multiple comparison using the false discovery rate approach.
