# Supplementary material for: Prenatal tobacco exposure on brain morphometry partially mediated poor cognitive performance in preadolescent children
Source: NeuroImmune Pharm Ther. 2023 Jul 13;2(4):375–86. doi: 10.1515/nipt-2023-0013 (PMC10696570; doi:10.1515/nipt-2023-0013)
Supplement: Supplementary file 2 — Supplementary Material Details [file j_nipt-2023-0013_suppl_002.pdf]

Supplemental Table 5A. Meditation analysis of the effect of prenatal tobacco exposure (PTE) on composite scores (uncorrected standard scores in the NIH Toolbox), N=10,123.

Morphometric	Effect	Total Score composite		Fluid Intelligence Composite		Crystallized intelligence Composite	
measures	measures	Effect (95% C.I.)	LMM-p	Effect (95% C.I.)	LMM-p	Effect (95% C.I.)	LMM-p
Cortical thickness							
Parahippocampal	AME	-0.03 (-0.05, -0.01)	<0.001	-0.03 (-0.06, -0.01)	0.01	-0.02 (-0.04, -0.01)	0.005
Parahippocampal	ADE	-0.95 (-1.49, -0.42)	0.003	-1.08 (-1.79, -0.35)	0.003	-0.56 (-1, -0.12)	0.012
Parahippocampal	Total	-0.98 (-1.53, -0.45)	0.003	-1.11 (-1.81, -0.38)	0.003	-0.58 (-1.02, -0.15)	0.009
Parahippocampal	Proportion (in %)	3.1 (1.2, 7.9)	0.009	2.7 (0.8, 9.5)	0.019	3.4 (1.1, 12.8)	0.024
Cortical surface areas							
Total surface	AME	-0.12 (-0.19, -0.05)	<0.001	-0.09 (-0.15, -0.04)	<0.001	-0.11 (-0.17, -0.05)	<0.001
Total surface	ADE	-0.85 (-1.39, -0.31)	0.006	-1.02 (-1.74, -0.37)	0.006	-0.47 (-0.9, -0.06)	0.017
Total surface	Total	-0.97 (-1.51, -0.42)	<0.001	-1.11 (-1.85, -0.45)	0.003	-0.58 (-1.02, -0.16)	0.007
Total surface	Proportion (in %)	12.1 (5, 28.3)	<0.001	8.1 (3.4, 20.6)	0.009	19.5 (7.8, 61.1)	0.019
Caudal ACC	AME	-0.04 (-0.08, -0.01)	0.022	-0.03 (-0.07, 0)	0.037	-0.03 (-0.06, -0.01)	0.029
Caudal ACC	ADE	-0.92 (-1.5, -0.37)	<0.001	-1.06 (-1.76, -0.41)	0.008	-0.55 (-0.97, -0.12)	0.01
Caudal ACC	Total	-0.96 (-1.53, -0.43)	<0.001	-1.1 (-1.79, -0.45)	0.005	-0.58 (-1, -0.15)	0.007
Caudal ACC	Proportion (in %)	4.1 (0.9, 11.4)	0.024	2.9 (0.4, 9.8)	0.044	5.3 (1.1, 20.2)	0.04
Caudal Midfrontal	AME	-0.06 (-0.1, -0.02)	0.019	-0.04 (-0.08, -0.01)	0.022	-0.05 (-0.09, -0.01)	0.022
Caudal Midfrontal	ADE	-0.92 (-1.46, -0.38)	0.003	-1.04 (-1.7, -0.44)	<0.001	-0.52 (-0.93, -0.13)	0.013
Caudal Midfrontal	Total	-0.98 (-1.51, -0.42)	0.003	-1.08 (-1.74, -0.48)	<0.001	-0.57 (-0.97, -0.17)	0.01
Caudal Midfrontal	Proportion (in %)	5.7 (1.4, 14.9)	0.024	4 (0.9, 10.8)	0.024	8.4 (2.1, 26.8)	0.04
Entorhinal	AME	-0.04 (-0.07, -0.02)	0.005	-0.03 (-0.06, -0.01)	<0.001	-0.04 (-0.07, -0.02)	<0.001
Entorhinal	ADE	-0.92 (-1.48, -0.37)	0.006	-1.07 (-1.78, -0.37)	0.003	-0.53 (-0.96, -0.12)	0.019
Entorhinal	Total	-0.96 (-1.53, -0.41)	0.005	-1.1 (-1.82, -0.39)	<0.001	-0.57 (-1, -0.17)	0.01
Entorhinal	Proportion (in %)	4.3 (1.4, 11.1)	0.019	2.6 (0.7, 9)	<0.001	7.4 (2.5, 26)	0.024
Fusiform	AME	-0.05 (-0.1, 0)	0.049	-0.03 (-0.07, 0)	0.069	-0.05 (-0.09, 0)	0.065
Fusiform	ADE	-0.93 (-1.5, -0.4)	<0.001	-1.08 (-1.75, -0.42)	<0.001	-0.54 (-0.96, -0.13)	0.013
Fusiform	Total	-0.98 (-1.54, -0.46)	<0.001	-1.11 (-1.77, -0.47)	<0.001	-0.59 (-1.02, -0.17)	0.009
Fusiform	Proportion (in %)	4.9 (0.4, 14.5)	0.051	3 (0, 9.7)	0.072	8.1 (0.2, 31.2)	0.077
Inferior parietal	AME	-0.08 (-0.13, -0.04)	0.005	-0.06 (-0.11, -0.03)	<0.001	-0.08 (-0.12, -0.04)	<0.001
Inferior parietal	ADE	-0.9 (-1.47, -0.36)	<0.001	-1.04 (-1.71, -0.34)	0.003	-0.51 (-0.93, -0.1)	0.021
Inferior parietal	Total	-0.98 (-1.53, -0.43)	<0.001	-1.1 (-1.79, -0.41)	0.003	-0.59 (-1, -0.18)	0.009
Inferior parietal	Proportion (in %)	8.4 (3.6, 20)	0.009	5.7 (2.4, 17.4)	0.009	12.9 (5.4, 44.9)	0.023

Lateral Occiptal	AME	-0.03 (-0.07, 0)	0.103	-0.02 (-0.05, 0)	0.082	-0.03 (-0.07, 0)	0.077
Lateral Occiptal	ADE	-0.95 (-1.49, -0.44)	0.006	-1.07 (-1.78, -0.39)	0.003	-0.55 (-0.98, -0.13)	0.01
Lateral Occiptal	Total	-0.99 (-1.53, -0.46)	0.003	-1.09 (-1.8, -0.41)	0.003	-0.58 (-1.01, -0.16)	0.009
Lateral Occiptal	Proportion (in %)	3.3 (-0.4, 9)	0.105	2.2 (-0.1, 7.2)	0.083	5.4 (-0.2, 20)	0.083
Lateral orbitofrontal	AME	-0.05 (-0.1, 0)	0.037	-0.05 (-0.09, -0.01)	0.037	-0.05 (-0.09, -0.01)	0.037
Lateral orbitofrontal	ADE	-0.91 (-1.4, -0.42)	0.003	-1.08 (-1.74, -0.42)	0.003	-0.53 (-0.96, -0.14)	0.003
Lateral orbitofrontal	Total	-0.96 (-1.46, -0.45)	<0.001	-1.12 (-1.79, -0.47)	<0.001	-0.58 (-1.02, -0.19)	0.005
Lateral orbitofrontal	Proportion (in %)	5.4 (0.4, 13.8)	0.049	4.1 (0.6, 11.2)	0.041	7.9 (1.2, 25.5)	0.044
Lingual	AME	-0.07 (-0.1, -0.03)	<0.001	-0.05 (-0.09, -0.03)	<0.001	-0.06 (-0.09, -0.03)	<0.001
Lingual	ADE	-0.9 (-1.46, -0.37)	<0.001	-1.05 (-1.72, -0.37)	0.003	-0.52 (-0.94, -0.12)	0.015
Lingual	Total	-0.97 (-1.53, -0.44)	<0.001	-1.11 (-1.78, -0.44)	0.003	-0.58 (-0.99, -0.18)	0.009
Lingual	Proportion (in %)	6.8 (3.2, 16.8)	<0.001	4.9 (2.2, 13.5)	0.009	9.5 (4.1, 31.5)	0.023
Paracentral	AME	-0.02 (-0.05, 0)	0.037	-0.01 (-0.03, 0)	0.127	-0.03 (-0.05, 0)	0.047
Paracentral	ADE	-0.96 (-1.51, -0.39)	0.003	-1.08 (-1.81, -0.32)	0.003	-0.55 (-0.99, -0.12)	0.012
Paracentral	Total	-0.98 (-1.53, -0.42)	0.003	-1.09 (-1.82, -0.32)	0.003	-0.57 (-1.02, -0.14)	0.009
Paracentral	Proportion (in %)	2.3 (0.3, 6.9)	0.041	0.9 (-0.1, 3.7)	0.13	5.1 (0.5, 18.3)	0.058
Pericalcarine	AME	-0.08 (-0.12, -0.05)	<0.001	-0.08 (-0.12, -0.04)	<0.001	-0.05 (-0.08, -0.03)	<0.001
Pericalcarine	ADE	-0.91 (-1.43, -0.39)	<0.001	-1.04 (-1.68, -0.35)	<0.001	-0.53 (-0.95, -0.13)	0.017
Pericalcarine	Total	-0.99 (-1.51, -0.46)	<0.001	-1.12 (-1.76, -0.45)	<0.001	-0.58 (-1, -0.17)	0.01
Pericalcarine	Proportion (in %)	7.9 (4.1, 17.4)	<0.001	7.3 (3.4, 18.4)	<0.001	9 (3.9, 30.6)	0.024
Posterior cingulate	AME	-0.07 (-0.11, -0.04)	<0.001	-0.05 (-0.08, -0.02)	<0.001	-0.07 (-0.1, -0.04)	<0.001
Posterior cingulate	ADE	-0.91 (-1.46, -0.39)	<0.001	-1.06 (-1.72, -0.36)	0.006	-0.51 (-0.94, -0.11)	0.017
Posterior cingulate	Total	-0.98 (-1.54, -0.46)	<0.001	-1.11 (-1.76, -0.41)	0.003	-0.58 (-1.02, -0.17)	0.009
Posterior cingulate	Proportion (in %)	7 (3.2, 16.4)	<0.001	4.3 (1.8, 12.9)	0.009	12 (5.3, 35.9)	0.023
Precentral	AME	-0.07 (-0.11, -0.03)	<0.001	-0.04 (-0.08, -0.02)	0.01	-0.07 (-0.11, -0.03)	0.005
Precentral	ADE	-0.92 (-1.46, -0.42)	<0.001	-1.05 (-1.69, -0.4)	<0.001	-0.5 (-0.89, -0.09)	0.013
Precentral	Total	-0.99 (-1.52, -0.46)	<0.001	-1.09 (-1.73, -0.43)	<0.001	-0.57 (-0.96, -0.15)	0.003
Precentral	Proportion (in %)	6.8 (2.3, 16.3)	<0.001	4.1 (1.3, 10.4)	0.015	11.9 (4.2, 48.2)	0.015
Rostral Midfrontal	AME	-0.1 (-0.16, -0.05)	<0.001	-0.07 (-0.11, -0.04)	<0.001	-0.11 (-0.16, -0.06)	<0.001
Rostral Midfrontal	ADE	-0.88 (-1.42, -0.34)	<0.001	-1.04 (-1.71, -0.38)	0.006	-0.48 (-0.91, -0.08)	0.023
Rostral Midfrontal	Total	-0.98 (-1.53, -0.45)	<0.001	-1.1 (-1.77, -0.45)	0.005	-0.59 (-1.02, -0.17)	0.009
Rostral Midfrontal	Proportion (in %)	10.5 (4.8, 25.6)	<0.001	6.2 (2.7, 16.8)	0.015	18.4 (8.2, 54.7)	0.023
Superior temporal	AME	-0.05 (-0.1, 0.01)	0.083	-0.03 (-0.07, 0)	0.082	-0.05 (-0.1, 0)	0.093
Superior temporal	ADE	-0.94 (-1.45, -0.41)	<0.001	-1.06 (-1.75, -0.43)	0.003	-0.53 (-0.95, -0.12)	0.015
Superior temporal	Total	-0.98 (-1.49, -0.46)	<0.001	-1.09 (-1.78, -0.45)	<0.001	-0.57 (-1, -0.16)	0.005
Superior temporal	Proportion (in %)	4.6 (-0.6, 14)	0.083	2.8 (-0.1, 8.7)	0.083	8.1 (-0.6, 29.6)	0.099

Supramarginal	AME	-0.04 (-0.07, 0)	0.083	-0.03 (-0.06, 0)	0.085	-0.03 (-0.07, 0)	0.087
Supramarginal	ADE	-0.96 (-1.49, -0.44)	<0.001	-1.06 (-1.75, -0.37)	0.006	-0.55 (-1, -0.11)	0.01
Supramarginal	Total	-0.99 (-1.52, -0.47)	<0.001	-1.08 (-1.77, -0.4)	0.005	-0.58 (-1.03, -0.15)	0.009
Supramarginal	Proportion (in %)	3.6 (-0.1, 9.6)	0.083	2.4 (-0.4, 8.5)	0.09	5.2 (-0.7, 18.7)	0.093
Temporal pole	AME	-0.03 (-0.06, -0.01)	0.022	-0.02 (-0.05, 0)	0.022	-0.03 (-0.06, -0.01)	0.015
Temporal pole	ADE	-0.95 (-1.48, -0.4)	<0.001	-1.1 (-1.77, -0.42)	<0.001	-0.54 (-0.96, -0.14)	0.015
Temporal pole	Total	-0.98 (-1.51, -0.44)	<0.001	-1.12 (-1.79, -0.43)	<0.001	-0.57 (-0.99, -0.16)	0.007
Temporal pole	Proportion (in %)	3.3 (0.7, 8.4)	0.024	1.8 (0.4, 5.8)	0.024	5.5 (1.4, 21.3)	0.028
Insula	AME	-0.05 (-0.09, -0.01)	0.039	-0.04 (-0.09, -0.01)	0.039	-0.04 (-0.07, -0.01)	0.022
Insula	ADE	-0.93 (-1.45, -0.38)	<0.001	-1.06 (-1.68, -0.36)	<0.001	-0.55 (-1.01, -0.16)	0.017
Insula	Total	-0.98 (-1.5, -0.43)	<0.001	-1.1 (-1.72, -0.4)	<0.001	-0.59 (-1.05, -0.19)	0.009
Insula	Proportion (in %)	4.8 (1, 13.1)	0.041	4 (0.6, 12.2)	0.041	6.2 (1.1, 19.3)	0.038
Postcentral	AME	-0.08 (-0.12, -0.04)	<0.001	-0.05 (-0.08, -0.02)	0.005	-0.08 (-0.12, -0.04)	<0.001
Postcentral	ADE	-0.91 (-1.44, -0.37)	<0.001	-1.04 (-1.73, -0.34)	0.003	-0.51 (-0.95, -0.08)	0.025
Postcentral	Total	-0.99 (-1.52, -0.44)	<0.001	-1.09 (-1.77, -0.4)	<0.001	-0.58 (-1.02, -0.16)	0.012
Postcentral	Proportion (in %)	7.6 (3.3, 19)	<0.001	4.5 (1.7, 13.6)	0.009	13.2 (5.3, 44)	0.028
Precuneus	AME	-0.03 (-0.08, 0.01)	0.143	-0.03 (-0.07, 0.01)	0.16	-0.03 (-0.06, 0.01)	0.149
Precuneus	ADE	-0.95 (-1.49, -0.4)	<0.001	-1.06 (-1.69, -0.37)	0.003	-0.56 (-0.97, -0.16)	0.01
Precuneus	Total	-0.98 (-1.53, -0.44)	<0.001	-1.09 (-1.74, -0.41)	0.003	-0.59 (-1, -0.19)	0.007
Precuneus	Proportion (in %)	3.3 (-0.8, 10.4)	0.143	2.8 (-0.9, 10)	0.163	4.3 (-1.6, 16.2)	0.156
Superior frontal	AME	-0.04 (-0.09, 0.01)	0.163	-0.03 (-0.07, 0.01)	0.204	-0.04 (-0.08, 0.02)	0.196
Superior frontal	ADE	-0.94 (-1.46, -0.43)	0.003	-1.07 (-1.76, -0.4)	0.006	-0.54 (-0.96, -0.15)	0.013
Superior frontal	Total	-0.98 (-1.51, -0.47)	0.003	-1.1 (-1.8, -0.44)	0.005	-0.58 (-1.01, -0.17)	0.007
Superior frontal	Proportion (in %)	3.9 (-1.5, 11.3)	0.166	2.6 (-1.2, 8.8)	0.209	6.1 (-3.2, 23.1)	0.198
Subcortical volumes							
Thalamus	AME	-0.09 (-0.14, -0.05)	<0.001	-0.09 (-0.14, -0.04)	<0.001	-0.06 (-0.09, -0.03)	<0.001
Thalamus	ADE	-0.82 (-1.38, -0.27)	0.003	-0.99 (-1.65, -0.33)	0.003	-0.44 (-0.87, -0.04)	0.032
Thalamus	Total	-0.91 (-1.48, -0.36)	<0.001	-1.07 (-1.73, -0.41)	<0.001	-0.5 (-0.94, -0.1)	0.012
Thalamus	Proportion (in %)	9.8 (4.7, 27.1)	<0.001	8 (3.5, 22.2)	<0.001	11.9 (5.1, 56.4)	0.028
Nucleus Accummbens	AME	-0.03 (-0.06, -0.01)	<0.001	-0.04 (-0.07, -0.01)	<0.001	-0.02 (-0.04, -0.01)	0.01
Nucleus Accummbens	ADE	-0.85 (-1.42, -0.33)	0.008	-1.01 (-1.69, -0.33)	<0.001	-0.44 (-0.83, -0.02)	0.038
Nucleus Accummbens	Total	-0.88 (-1.46, -0.36)	0.007	-1.05 (-1.73, -0.37)	<0.001	-0.46 (-0.85, -0.04)	0.026
Nucleus Accummbens	Proportion (in %)	3.9 (1.4, 11.4)	0.019	3.7 (1.1, 11.4)	<0.001	4.4 (0.8, 28.8)	0.051
Amygdala	AME	-0.01 (-0.03, 0.01)	0.209	0 (-0.02, 0.02)	0.938	-0.02 (-0.03, 0)	0.029
Amygdala	ADE	-0.85 (-1.38, -0.34)	<0.001	-1.03 (-1.73, -0.36)	<0.001	-0.45 (-0.85, -0.04)	0.04

Amygdala	Total	-0.86 (-1.4, -0.35)	<0.001	-1.03 (-1.73, -0.35)	<0.001	-0.46 (-0.87, -0.06)	0.034
Amygdala	Proportion (in %)	1.2 (-0.7, 5)	0.209	0.1 (-2.8, 2.7)	0.938	3.3 (0.1, 16.4)	0.074
Globus pallidum	AME	-0.04 (-0.07, -0.02)	<0.001	-0.04 (-0.07, -0.01)	<0.001	-0.03 (-0.06, -0.02)	<0.001
Globus pallidum	ADE	-0.86 (-1.4, -0.29)	0.003	-0.99 (-1.69, -0.31)	0.008	-0.44 (-0.86, -0.03)	0.036
Globus pallidum	Total	-0.91 (-1.45, -0.34)	0.003	-1.03 (-1.73, -0.35)	0.005	-0.48 (-0.89, -0.05)	0.026
Globus pallidum	Proportion (in %)	4.5 (1.9, 13.1)	0.009	3.6 (1.1, 11.2)	0.015	7 (2.4, 37.7)	0.047
Hippocampus	AME	-0.04 (-0.07, -0.02)	0.005	-0.03 (-0.07, -0.01)	<0.001	-0.04 (-0.06, -0.02)	<0.001
Hippocampus	ADE	-0.83 (-1.32, -0.29)	<0.001	-1.02 (-1.64, -0.34)	0.008	-0.45 (-0.86, -0.06)	0.023
Hippocampus	Total	-0.87 (-1.36, -0.33)	<0.001	-1.05 (-1.67, -0.38)	0.007	-0.49 (-0.9, -0.1)	0.012
Hippocampus	Proportion (in %)	4.8 (1.9, 13.7)	0.009	3.2 (0.9, 9.5)	0.019	7.5 (2.6, 35.5)	0.028
Putamen	AME	-0.02 (-0.05, 0)	0.047	-0.02 (-0.05, 0)	0.055	-0.02 (-0.04, 0)	0.057
Putamen	ADE	-0.87 (-1.39, -0.34)	<0.001	-1.03 (-1.76, -0.37)	0.003	-0.47 (-0.87, -0.04)	0.032
Putamen	Total	-0.89 (-1.42, -0.37)	<0.001	-1.05 (-1.79, -0.39)	0.003	-0.49 (-0.9, -0.06)	0.026
Putamen	Proportion (in %)	2.6 (0.3, 8.4)	0.049	2.1 (0.2, 7.6)	0.058	3.4 (-0.1, 14.6)	0.083

Supplemental Table 5A. Shows the effects of PTE on composite scores from the NIH Toolbox cognitive battery that showed a significant mediation by morphometric brain measures. The mediation effect estimates are presented with their 95% confidence intervals in parentheses. Mediation analyses were performed for composite scores and morphometric brain measures that showed a significant association with PTE in our cognitive performance and brain measure analyses after adjustment for covariates. Confidence intervals were generated using the quasi-Bayesian approach. All *p*-values were corrected for multiple comparison using the false discovery rate approach. From the sample used in previous steps in our analyses (N= 11,609), 10,123 children had complete information on cognitive performance and brain morphometry.

Abbreviations: AME = average mediated effect of PTE by brain morphometry. ADE = average direct effect of PTE; Total = total effect of PTE. Proportion = the proportion of the effect of PTE that is mediated by brain morphometry shown in. percentages.
