# Supplementary material for: Prenatal tobacco exposure on brain morphometry partially mediated poor cognitive performance in preadolescent children
Source: NeuroImmune Pharm Ther. 2023 Jul 13;2(4):375–86. doi: 10.1515/nipt-2023-0013 (PMC10696570; doi:10.1515/nipt-2023-0013)
Supplement: Supplementary file 3 — Supplementary Material Details [file j_nipt-2023-0013_suppl_003.pdf]

Supplemental Table 5B. Meditation analysis of the effect of prenatal tobacco exposure (PTE) on specific test scores (uncorrected standard scores in the NIH Toolbox), N=10,123.

Morphometric measures	Effect measures	Oral reading recognition		List sorting working memory		Picture sequence memory		Dimensional change card sort	
		Effect (95% C.I.)	LMM-p	Effect (95% C.I.)	LMM-p	Effect (95% C.I.)	LMM-p	Effect (95% C.I.)	LMM-p
Cortical thickness									
Parahippocampal	AME	-0.02 (-0.04, 0)	0.029	-0.01 (-0.04, 0.01)	0.273	-0.03 (-0.06, -0.01)	0.022	0 (-0.02, 0.01)	0.666
Parahippocampal	ADE	-0.67 (-1.08, -0.21)	0.003	-1.71 (-2.51, -0.94)	<0.001	-1.55 (-2.4, -0.69)	<0.001	-0.93 (-1.57, -0.33)	<0.001
Parahippocampal	Total	-0.68 (-1.11, -0.23)	0.003	-1.72 (-2.52, -0.97)	<0.001	-1.58 (-2.44, -0.72)	<0.001	-0.94 (-1.57, -0.33)	<0.001
Parahippocampal	Proportion (in %)	2.5 (0.5, 9.7)	0.034	0.8 (-0.6, 2.7)	0.273	2 (0.4, 5.3)	0.024	0.5 (-2, 3.5)	0.666
Cortical surface areas									
Total surface	AME	-0.1 (-0.16, -0.05)	<0.001	-0.15 (-0.23, -0.07)	<0.001	-0.02 (-0.05, 0)	0.132	-0.05 (-0.09, -0.02)	<0.001
Total surface	ADE	-0.59 (-1.02, -0.18)	0.012	-1.61 (-2.36, -0.88)	<0.001	-1.56 (-2.43, -0.8)	0.003	-0.86 (-1.46, -0.21)	0.015
Total surface	Total	-0.69 (-1.12, -0.28)	0.005	-1.76 (-2.53, -1.04)	<0.001	-1.58 (-2.45, -0.8)	0.003	-0.91 (-1.51, -0.26)	0.01
Total surface	Proportion (in %)	14.7 (6.4, 35.8)	0.015	8.3 (3.7, 16.5)	<0.001	1.1 (-0.3, 3.5)	0.134	5.6 (2.1, 18.9)	0.024
Caudal ACC	AME	-0.03 (-0.05, 0)	0.042	-0.04 (-0.09, 0)	0.055	-0.02 (-0.05, 0)	0.037	-0.02 (-0.04, 0)	0.025
Caudal ACC	ADE	-0.66 (-1.09, -0.21)	<0.001	-1.68 (-2.47, -0.92)	<0.001	-1.56 (-2.4, -0.65)	0.003	-0.9 (-1.57, -0.27)	0.008
Caudal ACC	Total	-0.69 (-1.12, -0.24)	<0.001	-1.73 (-2.51, -0.95)	<0.001	-1.59 (-2.43, -0.68)	0.003	-0.92 (-1.58, -0.29)	0.005
Caudal ACC	Proportion (in %)	3.9 (0.6, 12.7)	0.044	2.5 (0.2, 6)	0.056	1.4 (0.2, 4.6)	0.041	1.7 (0.2, 6.5)	0.034
Caudal Midfrontal	AME	-0.04 (-0.08, -0.01)	0.015	-0.07 (-0.13, -0.02)	0.005	-0.01 (-0.03, 0.01)	0.459	-0.03 (-0.05, -0.01)	0.025
Caudal Midfrontal	ADE	-0.64 (-1.11, -0.22)	0.006	-1.63 (-2.45, -0.88)	<0.001	-1.58 (-2.37, -0.79)	<0.001	-0.92 (-1.59, -0.28)	0.008
Caudal Midfrontal	Total	-0.69 (-1.15, -0.27)	0.005	-1.71 (-2.52, -0.95)	<0.001	-1.58 (-2.37, -0.79)	<0.001	-0.94 (-1.62, -0.31)	0.005
Caudal Midfrontal	Proportion (in %)	6.3 (1.8, 19.4)	0.024	4.3 (1.1, 10.5)	0.009	0.3 (-0.7, 2.1)	0.459	2.8 (0.5, 8.7)	0.034
Entorhinal	AME	-0.05 (-0.08, -0.02)	<0.001	-0.06 (-0.1, -0.02)	<0.001	0 (-0.03, 0.02)	0.776	-0.02 (-0.04, 0)	0.077
Entorhinal	ADE	-0.64 (-1.09, -0.22)	0.006	-1.66 (-2.43, -0.84)	<0.001	-1.58 (-2.4, -0.72)	<0.001	-0.9 (-1.51, -0.29)	0.003
Entorhinal	Total	-0.69 (-1.14, -0.26)	0.003	-1.71 (-2.48, -0.89)	<0.001	-1.59 (-2.41, -0.73)	<0.001	-0.91 (-1.51, -0.31)	<0.001
Entorhinal	Proportion (in %)	6.7 (2.5, 18)	0.009	3.2 (1, 7.2)	<0.001	0.2 (-1.4, 2)	0.774	1.6 (0, 6.4)	0.079
Fusiform	AME	-0.04 (-0.09, 0)	0.069	-0.06 (-0.12, 0)	0.055	0 (-0.02, 0.01)	0.476	-0.02 (-0.05, 0)	0.069
Fusiform	ADE	-0.66 (-1.09, -0.21)	<0.001	-1.64 (-2.43, -0.86)	<0.001	-1.59 (-2.48, -0.74)	<0.001	-0.92 (-1.57, -0.3)	0.012
Fusiform	Total	-0.7 (-1.15, -0.25)	<0.001	-1.7 (-2.51, -0.91)	<0.001	-1.6 (-2.48, -0.75)	<0.001	-0.94 (-1.61, -0.32)	0.009
Fusiform	Proportion (in %)	6.1 (0.1, 18.6)	0.072	3.5 (0.2, 8.6)	0.056	0.2 (-0.5, 1.6)	0.476	2.4 (0, 8.2)	0.081
Inferior parietal	AME	-0.07 (-0.11, -0.03)	0.005	-0.11 (-0.18, -0.05)	<0.001	-0.02 (-0.05, 0)	0.105	-0.04 (-0.07, -0.02)	<0.001
Inferior parietal	ADE	-0.63 (-1.08, -0.2)	0.003	-1.6 (-2.41, -0.74)	<0.001	-1.57 (-2.41, -0.77)	<0.001	-0.89 (-1.53, -0.25)	0.008
Inferior parietal	Total	-0.7 (-1.14, -0.27)	0.003	-1.71 (-2.52, -0.87)	<0.001	-1.59 (-2.43, -0.78)	<0.001	-0.93 (-1.57, -0.29)	0.003
Inferior parietal	Proportion (in %)	9.9 (4, 25.8)	0.015	6.4 (2.8, 13.5)	<0.001	1.1 (-0.2, 3.5)	0.105	4 (1.5, 13.1)	0.009

Lateral Occiptal	AME	-0.03 (-0.07, 0)	0.085	-0.04 (-0.09, 0)	0.082	0 (-0.01, 0.02)	0.498	-0.01 (-0.04, 0)	0.078
Lateral Occiptal	ADE	-0.66 (-1.12, -0.22)	<0.001	-1.67 (-2.47, -0.87)	<0.001	-1.58 (-2.45, -0.73)	<0.001	-0.92 (-1.51, -0.28)	0.008
Lateral Occiptal	Total	-0.69 (-1.15, -0.25)	<0.001	-1.71 (-2.5, -0.88)	<0.001	-1.57 (-2.45, -0.73)	<0.001	-0.93 (-1.53, -0.29)	0.005
Lateral Occiptal	Proportion (in %)	4.5 (-0.3, 14.5)	0.086	2.4 (-0.1, 6.4)	0.083	-0.2 (-1.8, 0.5)	0.498	1.4 (-0.1, 5.8)	0.083
Lateral orbitofrontal	AME	-0.04 (-0.08, -0.01)	0.037	-0.07 (-0.13, -0.01)	0.037	-0.01 (-0.04, 0)	0.037	-0.02 (-0.05, 0)	0.037
Lateral orbitofrontal	ADE	-0.67 (-1.1, -0.19)	0.003	-1.64 (-2.4, -0.84)	0.003	-1.55 (-2.43, -0.71)	0.003	-0.9 (-1.5, -0.25)	0.003
Lateral orbitofrontal	Total	-0.71 (-1.14, -0.24)	<0.001	-1.71 (-2.51, -0.91)	<0.001	-1.57 (-2.45, -0.72)	<0.001	-0.92 (-1.52, -0.27)	0.007
Lateral orbitofrontal	Proportion (in %)	5.4 (0.8, 18.6)	0.04	4.2 (0.7, 9.6)	0.034	0.9 (0, 2.9)	0.072	2.5 (0.1, 9.2)	0.072
Lingual	AME	-0.06 (-0.09, -0.03)	<0.001	-0.07 (-0.11, -0.03)	<0.001	0 (-0.03, 0.03)	0.876	-0.04 (-0.07, -0.02)	<0.001
Lingual	ADE	-0.63 (-1.06, -0.23)	0.006	-1.64 (-2.43, -0.91)	<0.001	-1.57 (-2.43, -0.72)	<0.001	-0.88 (-1.52, -0.29)	0.01
Lingual	Total	-0.69 (-1.11, -0.28)	<0.001	-1.71 (-2.49, -0.96)	<0.001	-1.57 (-2.43, -0.71)	<0.001	-0.92 (-1.56, -0.33)	0.007
Lingual	Proportion (in %)	8.2 (3.8, 21.9)	<0.001	3.7 (1.7, 7.9)	<0.001	-0.1 (-2.2, 1.8)	0.876	4.3 (1.3, 12.7)	0.019
Paracentral	AME	-0.03 (-0.05, 0)	0.045	-0.03 (-0.07, -0.01)	0.037	0.01 (0, 0.03)	0.228	0 (-0.01, 0.02)	0.683
Paracentral	ADE	-0.67 (-1.12, -0.25)	0.008	-1.68 (-2.52, -0.88)	<0.001	-1.58 (-2.37, -0.77)	<0.001	-0.92 (-1.59, -0.24)	0.006
Paracentral	Total	-0.7 (-1.15, -0.28)	0.003	-1.72 (-2.55, -0.91)	<0.001	-1.57 (-2.37, -0.76)	<0.001	-0.92 (-1.58, -0.24)	0.005
Paracentral	Proportion (in %)	3.7 (0.5, 11.4)	0.049	1.9 (0.3, 4.8)	0.04	-0.5 (-2.3, 0.2)	0.228	-0.2 (-2.5, 1.2)	0.683
Pericalcarine	AME	-0.05 (-0.07, -0.03)	<0.001	-0.09 (-0.14, -0.05)	<0.001	-0.02 (-0.06, 0.01)	0.182	-0.06 (-0.1, -0.03)	<0.001
Pericalcarine	ADE	-0.65 (-1.12, -0.22)	0.006	-1.63 (-2.51, -0.84)	<0.001	-1.57 (-2.4, -0.69)	0.006	-0.86 (-1.49, -0.26)	0.008
Pericalcarine	Total	-0.69 (-1.16, -0.27)	0.003	-1.72 (-2.63, -0.93)	<0.001	-1.59 (-2.43, -0.73)	0.003	-0.92 (-1.55, -0.33)	0.005
Pericalcarine	Proportion (in %)	6.6 (3, 18.3)	0.009	5.2 (2.6, 11)	<0.001	1.4 (-0.6, 4.6)	0.184	6.7 (3.1, 21.1)	0.015
Posterior cingulate	AME	-0.07 (-0.1, -0.03)	<0.001	-0.08 (-0.13, -0.04)	<0.001	-0.02 (-0.04, 0.01)	0.246	-0.03 (-0.05, -0.01)	0.015
Posterior cingulate	ADE	-0.62 (-1.06, -0.21)	0.003	-1.61 (-2.4, -0.8)	<0.001	-1.58 (-2.37, -0.76)	<0.001	-0.91 (-1.58, -0.24)	0.012
Posterior cingulate	Total	-0.69 (-1.13, -0.28)	<0.001	-1.69 (-2.48, -0.89)	<0.001	-1.59 (-2.4, -0.78)	<0.001	-0.94 (-1.61, -0.27)	0.009
Posterior cingulate	Proportion (in %)	9.5 (4.6, 25.7)	<0.001	4.7 (2.1, 10.4)	<0.001	1 (-0.6, 3.6)	0.246	2.8 (0.7, 10.1)	0.032
Precentral	AME	-0.06 (-0.1, -0.02)	0.005	-0.09 (-0.15, -0.03)	<0.001	0 (-0.02, 0.02)	0.616	-0.02 (-0.05, -0.01)	0.005
Precentral	ADE	-0.63 (-1.06, -0.22)	0.006	-1.62 (-2.46, -0.84)	<0.001	-1.6 (-2.42, -0.79)	<0.001	-0.89 (-1.56, -0.26)	<0.001
Precentral	Total	-0.7 (-1.12, -0.28)	0.003	-1.7 (-2.56, -0.93)	<0.001	-1.6 (-2.42, -0.78)	<0.001	-0.91 (-1.58, -0.29)	<0.001
Precentral	Proportion (in %)	8.8 (3, 22.2)	0.015	5 (2, 11)	<0.001	-0.3 (-1.8, 1)	0.616	2.6 (0.7, 10.5)	0.009
Rostral Midfrontal	AME	-0.1 (-0.15, -0.05)	<0.001	-0.14 (-0.21, -0.08)	<0.001	-0.02 (-0.04, 0.01)	0.233	-0.04 (-0.06, -0.01)	0.005
Rostral Midfrontal	ADE	-0.6 (-1.05, -0.15)	0.006	-1.58 (-2.34, -0.78)	<0.001	-1.58 (-2.4, -0.72)	<0.001	-0.87 (-1.5, -0.28)	0.006
Rostral Midfrontal	Total	-0.69 (-1.14, -0.24)	0.003	-1.71 (-2.47, -0.92)	<0.001	-1.6 (-2.41, -0.74)	<0.001	-0.91 (-1.54, -0.31)	0.005
Rostral Midfrontal	Proportion (in %)	14 (6.7, 36.9)	0.009	8 (3.9, 17)	<0.001	0.9 (-0.7, 3.7)	0.233	3.8 (1.3, 13)	0.019
Superior temporal	AME	-0.04 (-0.09, 0)	0.092	-0.06 (-0.12, 0)	0.083	0 (-0.02, 0.01)	0.556	-0.02 (-0.04, 0)	0.078
Superior temporal	ADE	-0.65 (-1.11, -0.2)	0.003	-1.68 (-2.51, -0.89)	<0.001	-1.59 (-2.46, -0.78)	<0.001	-0.92 (-1.54, -0.29)	0.008
Superior temporal	Total	-0.69 (-1.15, -0.23)	0.003	-1.74 (-2.56, -0.94)	<0.001	-1.6 (-2.46, -0.79)	<0.001	-0.93 (-1.55, -0.3)	0.007
Superior temporal	Proportion (in %)	5.8 (-0.5, 20.8)	0.094	3.5 (-0.1, 8.9)	0.083	0.2 (-0.5, 1.4)	0.556	1.5 (-0.1, 5.6)	0.083

Supramarginal	AME	-0.03 (-0.06, 0)	0.077	-0.05 (-0.1, 0)	0.093	-0.01 (-0.03, 0)	0.176	-0.02 (-0.04, 0)	0.107
Supramarginal	ADE	-0.67 (-1.09, -0.19)	0.01	-1.69 (-2.48, -0.91)	<0.001	-1.58 (-2.47, -0.78)	<0.001	-0.9 (-1.53, -0.28)	0.008
Supramarginal	Total	-0.7 (-1.13, -0.22)	0.009	-1.74 (-2.52, -0.98)	<0.001	-1.59 (-2.48, -0.78)	<0.001	-0.91 (-1.55, -0.29)	0.007
Supramarginal	Proportion (in %)	4 (-0.3, 13.4)	0.083	2.6 (-0.1, 7)	0.094	0.5 (-0.2, 2)	0.176	1.7 (-0.2, 7.5)	0.115
Temporal pole	AME	-0.03 (-0.05, -0.01)	0.032	-0.05 (-0.1, -0.02)	0.005	0.01 (-0.01, 0.02)	0.481	-0.01 (-0.02, 0)	0.172
Temporal pole	ADE	-0.68 (-1.13, -0.22)	0.008	-1.64 (-2.43, -0.89)	<0.001	-1.61 (-2.51, -0.74)	<0.001	-0.93 (-1.6, -0.28)	0.006
Temporal pole	Total	-0.7 (-1.16, -0.26)	0.007	-1.69 (-2.47, -0.94)	<0.001	-1.6 (-2.51, -0.73)	<0.001	-0.94 (-1.61, -0.3)	0.005
Temporal pole	Proportion (in %)	3.9 (0.9, 11.8)	0.041	3 (0.9, 7.1)	0.009	-0.3 (-1.7, 0.7)	0.481	0.8 (-0.4, 3.9)	0.174
Insula	AME	-0.03 (-0.06, 0)	0.047	-0.07 (-0.13, -0.01)	0.032	0 (-0.02, 0.01)	0.572	-0.03 (-0.07, 0)	0.047
Insula	ADE	-0.66 (-1.09, -0.22)	0.008	-1.64 (-2.41, -0.83)	<0.001	-1.55 (-2.4, -0.7)	<0.001	-0.88 (-1.56, -0.3)	0.003
Insula	Total	-0.69 (-1.12, -0.24)	0.005	-1.71 (-2.47, -0.9)	<0.001	-1.55 (-2.41, -0.71)	<0.001	-0.91 (-1.6, -0.34)	0.003
Insula	Proportion (in %)	4.8 (0.4, 13.7)	0.054	3.9 (0.6, 9.1)	0.034	0.2 (-0.7, 1.6)	0.572	3.5 (0.4, 12.7)	0.051
Postcentral	AME	-0.08 (-0.12, -0.04)	<0.001	-0.09 (-0.15, -0.05)	<0.001	0 (-0.03, 0.02)	0.764	-0.03 (-0.06, -0.01)	<0.001
Postcentral	ADE	-0.63 (-1.06, -0.2)	0.006	-1.62 (-2.39, -0.81)	<0.001	-1.59 (-2.46, -0.74)	<0.001	-0.89 (-1.49, -0.24)	0.006
Postcentral	Total	-0.71 (-1.15, -0.27)	<0.001	-1.71 (-2.49, -0.93)	<0.001	-1.59 (-2.46, -0.75)	<0.001	-0.92 (-1.53, -0.29)	0.003
Postcentral	Proportion (in %)	11 (4.9, 26.9)	<0.001	5.3 (2.5, 11.9)	<0.001	0.2 (-1.5, 2.3)	0.764	3.8 (1.3, 13.4)	0.009
Precuneus	AME	-0.02 (-0.05, 0.01)	0.143	-0.04 (-0.09, 0.01)	0.156	-0.01 (-0.03, 0)	0.156	-0.02 (-0.04, 0)	0.162
Precuneus	ADE	-0.69 (-1.14, -0.25)	0.003	-1.67 (-2.49, -0.87)	<0.001	-1.58 (-2.39, -0.73)	0.003	-0.91 (-1.6, -0.33)	0.008
Precuneus	Total	-0.71 (-1.16, -0.28)	0.003	-1.71 (-2.52, -0.91)	<0.001	-1.59 (-2.41, -0.75)	0.003	-0.93 (-1.6, -0.34)	0.007
Precuneus	Proportion (in %)	3.2 (-0.9, 10.7)	0.145	2.2 (-0.7, 6.3)	0.156	0.6 (-0.2, 2.6)	0.159	1.9 (-0.5, 7.6)	0.164
Superior frontal	AME	-0.03 (-0.07, 0.01)	0.174	-0.05 (-0.11, 0.02)	0.188	-0.01 (-0.02, 0)	0.374	-0.02 (-0.05, 0.01)	0.205
Superior frontal	ADE	-0.67 (-1.11, -0.23)	0.003	-1.67 (-2.42, -0.9)	<0.001	-1.58 (-2.36, -0.75)	<0.001	-0.92 (-1.55, -0.31)	0.008
Superior frontal	Total	-0.7 (-1.15, -0.26)	0.003	-1.72 (-2.47, -0.96)	<0.001	-1.58 (-2.37, -0.75)	<0.001	-0.94 (-1.57, -0.33)	0.005
Superior frontal	Proportion (in %)	4.5 (-1.9, 17.4)	0.173	2.7 (-1.3, 7.1)	0.188	0.2 (-0.3, 1.4)	0.374	1.8 (-0.8, 7.7)	0.209
Subcortical volumes									
Thalamus	AME	-0.04 (-0.06, -0.02)	<0.001	-0.07 (-0.12, -0.03)	<0.001	-0.03 (-0.08, 0.01)	0.173	-0.07 (-0.11, -0.03)	<0.001
Thalamus	ADE	-0.55 (-0.98, -0.14)	0.006	-1.56 (-2.39, -0.76)	<0.001	-1.55 (-2.39, -0.71)	<0.001	-0.86 (-1.55, -0.24)	0.013
Thalamus	Total	-0.59 (-1.03, -0.16)	0.005	-1.62 (-2.46, -0.84)	<0.001	-1.58 (-2.43, -0.73)	<0.001	-0.92 (-1.61, -0.3)	0.005
Thalamus	Proportion (in %)	6.4 (2.1, 21.6)	0.015	4.1 (1.5, 9.8)	<0.001	1.8 (-0.7, 6)	0.173	7.4 (3, 22.4)	0.015
Nucleus Accummbens	AME	-0.02 (-0.03, 0)	0.045	-0.03 (-0.07, -0.01)	0.037	-0.04 (-0.08, -0.01)	0.015	-0.03 (-0.06, -0.01)	0.019
Nucleus Accummbens	ADE	-0.57 (-0.99, -0.14)	0.008	-1.61 (-2.41, -0.8)	<0.001	-1.54 (-2.33, -0.67)	<0.001	-0.85 (-1.48, -0.17)	0.013
Nucleus Accummbens	Total	-0.59 (-1.01, -0.16)	0.005	-1.64 (-2.43, -0.83)	<0.001	-1.58 (-2.37, -0.7)	<0.001	-0.88 (-1.51, -0.2)	0.012
Nucleus Accummbens	Proportion (in %)	2.8 (0.2, 11.3)	0.051	1.8 (0.3, 4.8)	0.04	2.7 (0.8, 7.6)	0.019	3.6 (0.6, 14.1)	0.04
Amygdala	AME	-0.02 (-0.04, 0)	0.022	0.01 (-0.02, 0.03)	0.573	0.01 (-0.01, 0.04)	0.363	-0.01 (-0.03, 0.01)	0.383
Amygdala	ADE	-0.59 (-1.03, -0.17)	0.003	-1.61 (-2.43, -0.82)	<0.001	-1.59 (-2.47, -0.81)	<0.001	-0.89 (-1.47, -0.28)	0.003

Amygdala	Total	-0.61 (-1.05, -0.19)	<0.001	-1.6 (-2.42, -0.8)	<0.001	-1.58 (-2.46, -0.79)	<0.001	-0.9 (-1.48, -0.28)	0.003
Amygdala	Proportion (in %)	2.8 (0.6, 11.3)	0.024	-0.5 (-2.6, 1.3)	0.573	-0.8 (-3.3, 0.8)	0.363	1.1 (-1.1, 5)	0.381
Globus pallidum	AME	-0.03 (-0.06, -0.01)	<0.001	-0.02 (-0.05, 0.01)	0.156	0 (-0.03, 0.03)	0.776	-0.04 (-0.07, -0.01)	<0.001
Globus pallidum	ADE	-0.56 (-0.98, -0.11)	0.015	-1.59 (-2.39, -0.71)	<0.001	-1.57 (-2.45, -0.65)	0.003	-0.88 (-1.52, -0.22)	0.015
Globus pallidum	Total	-0.59 (-1.03, -0.14)	0.009	-1.61 (-2.4, -0.72)	<0.001	-1.57 (-2.45, -0.66)	0.003	-0.91 (-1.56, -0.25)	0.009
Globus pallidum	Proportion (in %)	5.4 (1.9, 20.2)	0.023	1.3 (-0.4, 4.1)	0.157	0.2 (-1.8, 2.6)	0.774	4 (1.2, 14.4)	0.023
Hippocampus	AME	-0.03 (-0.05, -0.01)	<0.001	-0.01 (-0.04, 0.01)	0.409	-0.04 (-0.08, -0.01)	<0.001	-0.04 (-0.07, -0.01)	0.005
Hippocampus	ADE	-0.58 (-1.02, -0.17)	0.012	-1.58 (-2.38, -0.8)	<0.001	-1.56 (-2.4, -0.7)	<0.001	-0.87 (-1.54, -0.21)	0.013
Hippocampus	Total	-0.61 (-1.05, -0.19)	0.009	-1.59 (-2.39, -0.83)	<0.001	-1.6 (-2.44, -0.75)	<0.001	-0.91 (-1.57, -0.24)	0.009
Hippocampus	Proportion (in %)	4.8 (1.5, 16.1)	0.023	0.6 (-0.8, 2.9)	0.409	2.5 (0.8, 7)	<0.001	4.1 (1.3, 13.6)	0.024
Putamen	AME	-0.02 (-0.04, 0)	0.057	-0.01 (-0.03, 0.01)	0.316	0.01 (-0.01, 0.03)	0.409	-0.02 (-0.05, 0)	0.037
Putamen	ADE	-0.58 (-1.01, -0.13)	0.013	-1.59 (-2.36, -0.81)	<0.001	-1.59 (-2.37, -0.73)	<0.001	-0.88 (-1.54, -0.26)	0.01
Putamen	Total	-0.6 (-1.04, -0.16)	0.007	-1.6 (-2.37, -0.82)	<0.001	-1.58 (-2.37, -0.72)	<0.001	-0.9 (-1.56, -0.29)	0.007
Putamen	Proportion (in %)	3.2 (0.2, 14.8)	0.067	0.4 (-0.4, 2)	0.316	-0.3 (-2.1, 0.5)	0.409	2.7 (0.2, 9.8)	0.047

Supplemental Table 5B. Shows the effects of PTE on specific test scores from the NIH Toolbox cognitive battery that showed a significant mediation by morphometric measures. The mediation effect estimates are presented with their 95% confidence intervals in parentheses. Mediation analyses were performed for specific test scores and morphometric measures that showed a significant association with PTE in our cognitive performance and brain measure analyses after adjustment for covariates. Confidence intervals were generated using the quasi-Bayesian approach. All *p*-values were corrected for multiple comparison using the false discovery rate approach. From the sample used in previous steps in our analyses (N= 11,609), 10,123 children had complete information on cognitive performance and brain morphometry.

Abbreviations: AME = average mediated effect of PTE by brain morphometry. ADE = average direct effect of PTE; Total = total effect of PTE. Proportion = the proportion of the effect of PTE that is mediated by brain morphometry shown in. percentages.
